# Emissivity Correction Changes the Maintenance Decision: Reference-Free Validation of Material-Resolved Severity Grading in Proximal Thermal Remote Sensing of High-Voltage Substations

**DOI:** 10.3390/s26217007

**Published:** 2026-11-05

**Authors:** Irfan Ullah, Farhan Ullah, Khalil Khan, Rehan Ullah Khan, JaKeoung Koo

**Affiliations:** 1 School of Computer Science, Chengdu University of Technology, Chengdu 610059, China; irfan.ee@cqu.edu.cn; 2 School of Computing, Gachon University, Seongnam 13120, Republic of Korea; farhan.marwat@gmail.com (F.U.); jakeoung@gachon.ac.kr (J.K.); 3 Department of Information Technology, College of Computer, Qassim University, Buraydah 52571, Saudi Arabia; re.khan@qu.edu.sa

**Keywords:** infrared thermography, proximal remote sensing, emissivity correction, substation equipment, visible-infrared registration, image processing, computer vision, uncertainty propagation, Monte Carlo, machine learning, measurement uncertainty condition-based maintenance, ANSI/NETA MTS, reference-free validation

## Abstract

Proximal, ground-based infrared thermography of substation equipment grades a temperature rise computed under a single, camera-default emissivity and reflected-temperature setting applied to every pixel, regardless of the material it shows. This assumption is inexpensive but not harmless: two components at the same physical temperature but emissivities of 0.3 and 0.9 are read 24.0 K apart, enough to shift a component between adjacent bands of the ANSI/NETA MTS maintenance-priority scale. The measurement equation responsible sits on the same physical basis as land surface temperature retrieval from satellite and airborne thermal sensors, where emissivity is likewise the term most often assumed rather than measured. This paper corrects and validates that assumption at corpus scale by fusing the thermal frame with what a modern radiometric camera already records alongside it, a co-registered visible-light photograph, on a public 895-frame corpus of substation thermograms acquired under a single fixed set of acquisition parameters. The visible channel is used to rebuild the radiometric measurement equation, recover a per-frame reflected temperature from sky segmentation, and estimate in-situ emissivity at 106 metal-to-insulator material boundaries, with the resulting uncertainty propagated by a per-frame Monte Carlo to the severity band assigned to 765 graded frames. Three reference-free tests, a within-insulator negative control, a joint emissivity-reflectance fit, and a cross-view repeatability analysis, validate the estimated emissivity against physical constraints rather than a laboratory thermometer. Under the conventional absolute-temperature criterion, 48.4% of frames cannot be assigned a severity band with 80% confidence even once air temperature is obtained from meteorological reanalysis rather than a fixed default; grading instead against a photometrically similar in-frame reference surface reduces this to 5.9% (12.2% once the reference surface’s own sampling noise is additionally propagated through the same Monte Carlo). A value-of-information ranking identifies the single additional measurement, by material, that would most reduce this ambiguity. These results argue for relative grading criteria over a more accurate default emissivity table, an approach tied to the visible-thermal pairing common to modern radiometric cameras rather than to a specific camera model or acquisition site.

## 1. Introduction

High-voltage substations are critical nodes in electricity transmission and distribution networks, and unplanned failure of the equipment they contain, circuit breakers, disconnectors, surge arresters, wave traps and power transformers alike, can interrupt supply to large numbers of consumers, damage adjacent equipment through cascading heating or arcing, and endanger personnel working nearby. Condition-based maintenance, in which an intervention is prioritised by a measured indicator of equipment condition rather than by a fixed calendar interval, is the practice electric utilities use to manage this risk, and infrared thermography is its principal non-contact diagnostic tool [1,2,3]. In routine field use, a technician walks a substation with a handheld radiometric camera, and a temperature rise above ambient air or above a comparable phase is read against a maintenance-priority table such as ANSI/NETA MTS Table 100.18, which assigns increasing urgency to rises of approximately 1 to 10 K, 11 to 20 K, 21 to 40 K and above 40 K [1]. The temperature that a radiometric camera reports, however, is not a direct measurement. It is the solution of a measurement equation that separates the detector’s received radiance into three sources, the object itself, radiance reflected from the surroundings, and emission and absorption by the intervening atmosphere, weighted respectively by the surface emissivity ε, one minus that emissivity, and the atmospheric transmittance [4,5]. Two of the three terms in that equation, emissivity and reflected temperature, are set once per acquisition session by the camera operator and then held fixed for every pixel in every frame, independent of what surface the pixel shows.

This is a defensible simplification when the surface in front of the lens is uniform and known, and it is the practice that field thermography manuals and the camera manufacturers themselves recommend as a workable default [6]. It is a poor description of a substation, where a single wide field of view routinely contains glazed porcelain insulators (emissivity commonly reported at 0.90 to 0.95 [7]), painted steel and aluminium enclosures (0.85 to 0.95 when the paint is intact), galvanised steel (0.25 to 0.60 depending on weathering [8]) and bare or lightly oxidised metal fittings and conductors, for which reported emissivities span an unusually wide range: near 0.1 for clean aluminium in handbook tables, 0.25 to 0.45 in dynamic-line-rating studies of in-service conductors [9,10], 0.43 to 0.89 for used clamps, connectors and switch hardware measured directly in the 8 to 14 μm band [11], and 0.85 to 0.98 for heavily weathered outdoor metal [12]. A single default emissivity, applied without regard to which of these surfaces is being graded, does not produce a mildly imprecise temperature. At a true surface temperature of 60 °C, a part with the field-observed lower bound for weathered metal (ε = 0.3) and one with the upper bound for glazed porcelain (ε = 0.9) are read 24.0 K apart under identical camera settings, and 12.3 K apart at 40 °C; both differences straddle at least one boundary of the four-band ANSI/NETA severity scale used to decide whether a component is inspected again next cycle or de-energised immediately. The practical consequence is that the same measurement equation that makes radiometric thermography quantitative in principle can silently substitute the operator’s uniform assumption for the graded decision it is meant to inform.

The obstacle to correcting this in the field has been practical rather than conceptual: assigning the correct emissivity to a pixel requires knowing what material it shows, and until recently no consumer-grade radiometric camera recorded that information alongside the thermal frame. Modern compact thermal cameras partially close this gap by recording a co-registered visible-light photograph in every acquisition, intended by the manufacturer as a documentation aid rather than a measurement channel. This paper treats that visible channel as a measurement channel: it is used both to identify material at the graded location and to measure the reflected radiance the camera actually recorded, in place of the single value the operator set once for the whole session.

The idea of assigning a per-pixel or per-region emissivity from an auxiliary image rather than a single scene-wide constant has been explored across several domains. The most established precedent solves a structurally identical under-determined problem at a different scale: temperature-emissivity separation (TES) algorithms recover both temperature and emissivity from a single measured radiance using redundant spectral bands rather than an auxiliary image, first formulated for the Advanced Spaceborne Thermal Emission and Reflection Radiometer [13] and adapted since to other spaceborne thermal-infrared sensors [14]; the present paper instead resolves the same indeterminacy with a second, co-registered channel rather than additional spectral bands. Ding et al. [15] use visible-light segmentation to switch between tabulated emissivities in thermal imaging of electrical equipment, an early instance of the visible-guided correction strategy pursued here. Gao and Tian [16] and Schmid et al. [17] correct emissivity from spectral correlation between infrared and visible imagery and from a trained neural network, respectively, both without a substation-specific application. Two 2026 papers are the nearest prior work to the present study and are treated in detail below. Beyond correction strategies, recent work continues to refine how emissivity itself is estimated: Toullier et al. [18] jointly estimate emissivity and temperature with a Kriged marginalised particle filter; Rodríguez et al. [19] compare numerical and machine-learning routes to the same joint estimate; Mlačnik and Pušnik [20] compare radiometric and spectrometric emissivity evaluation directly; Ennass et al. [21] and Yang et al. [22] use multispectral and finite-element routes for high-temperature metallic surfaces; and Kim and Song [23] apply pixel-level emissivity correction to micrometre-scale operando thermography. None of these combines the correction with a validation that can fail on physical grounds in the absence of a reference thermometer, which is the central methodological contribution of the present paper.

Beyond emissivity itself, the reflected-temperature term is frequently assumed rather than measured, a simplification this paper’s own corpus exhibits in its most extreme form (a single fixed 20 °C for outdoor acquisitions ranging from about −22 °C to +21 °C in apparent sky temperature). The same term is handled at a much larger scale in land surface temperature retrieval from satellite and airborne thermal sensors, where separating emitted surface radiance from atmospheric downwelling and upwelling contributions without a coincident atmospheric sounding is a recurring difficulty [24]. Kruczek [25] and Toullier and Dumoulin [26] examine when a camera’s own sky-facing reading is usable as a reflected-temperature estimate outdoors, the same physical quantity measured here from the visible channel’s sky segmentation. Lafargue-Tallet et al. [27] use active thermo-reflectometry to separate reflected from emitted radiance on specular materials. Adán et al. [28] correct infrared camera temperatures for 3D building thermal models, and Silva Junior et al. [29] model atmospheric and load effects on substation equipment temperature specifically, the closest analogue in the literature to this paper’s own ambient severity model.

Any of these corrections depends on the visible channel first being properly aligned with the thermal frame, and registering a visible or RGB channel to a thermal frame in order to drive a downstream radiometric or detection task is well established for substations and for outdoor infrastructure generally. Zhang et al. [30] register infrared and visible images specifically for substations with a wide depth of field, the closest published registration setting to the one used here. Shao and Vasilev [31] and Güidi et al. [32] address calibration hardware and 3D thermal mapping for dual-sensor systems, and Meng et al. [33] register UAV-acquired thermal and visible pairs geometrically without a radiometric correction step.

Beyond registration and fusion, a parallel line of work applies semantic segmentation directly to substation and power-equipment imagery. Chen et al. [34] combine image splicing with semantic segmentation for omni-directional substation monitoring, and Liu et al. [35] propose a feature-differentiation fusion segmentation model for substation visual perception; Gomes et al. [36] release a 15-class annotated ground-truth dataset specifically for training such segmentation models on substation equipment. Liu et al. [37] and Wang et al. [38] report thermal-image segmentation methods and datasets for high-voltage equipment more broadly. None of this segmentation literature is radiometric: labels are used for detection or mapping, not to select a per-pixel emissivity, which is the role visible-channel segmentation plays in the present paper. Separately, UAV-based platforms extend inspection to line and tower infrastructure beyond what a handheld camera reaches: Li et al. [39] design an autonomous UAV inspection system for high-voltage transmission lines, and Chatzargyros et al. [40] report a field case study of AI-assisted UAV inspection combining structural, insulator and thermal fault detection. The same aerial thermal-sensing platform is established as a general-purpose remote-sensing instrument well beyond power engineering, evaluated directly against fixed-wing aircraft on the same physical basis for photovoltaic-plant inspection [41].

Closer to inspection practice than to image processing, Minkina and Gryś [2] discuss open problems in interpreting thermographic measurements in electrical power engineering, directly relevant to the correction problem addressed here. Zarco-Periñán et al. [42] propose a correction method for temperature problems specific to substation thermography, and a companion survey [43] documents inspection practice. Xia [3] reviews the state of the art in infrared diagnostics on power equipment broadly. Beyond the ANSI/NETA maintenance-priority table adopted here, ASTM [44] and ISO [45] set out general procedural guides for infrared inspection of electrical and mechanical equipment internationally, neither of which specifies how emissivity should be assigned at the graded point. None of this literature re-derives or replaces the NETA severity thresholds used operationally, nor propagates measurement uncertainty through to the banding decision, which this paper does directly.

None of this practice-oriented literature quantifies the uncertainty of the measurement itself, which a more statistical body of work addresses: concentrated around one research group, it applies Guide to the Expression of Uncertainty in Measurement (GUM)-style analysis to thermographic temperature measurement of electrical apparatus: Dziarski and Hulewicz [46,47] quantify uncertainty from an additional close-up lens and from switchgear-contained electric units; Hulewicz et al. [48] extend this to semiconductor junction temperature; García-López and Álvarez-Tey [49] evaluate surface-temperature uncertainty for photovoltaic modules outdoors. Ferreira et al. [50] analyse measurement uncertainty specifically in electrical-component infrared diagnosis, and Playà-Montmany and Tattersall [51] quantify spot-size, distance and emissivity errors in field applications of infrared thermography; both give an error model against which the uncertainty budget of Section 2.7 can be compared (Section 4.2). The Monte Carlo propagation itself follows the general procedure of GUM Supplement 1 [52], and the standardised-regression-coefficient sensitivity analysis is one of the variance-based approaches surveyed by Saltelli et al. [53] for apportioning output uncertainty among inputs. No prior study was found that combines a Monte Carlo uncertainty propagation with a standardised-regression-coefficient sensitivity analysis and a value-of-information ranking for thermographic maintenance decisions in the manner of Section 2.7, which this paper treats as a methodological contribution rather than a gap in the search.

Quantifying that uncertainty is one question; deciding which additional measurement would most reduce it is another. Ranking measurements this way, rather than reporting uncertainty for its own sake, has an established basis outside thermography. Sedivy et al. [54] review value-of-information approaches for prioritising road-infrastructure inspection and asset-management decisions, and Liu and Wang [55] develop a decision-theoretic framework for valuing a continuous monitoring system in recurrent maintenance scenarios. Neither addresses thermographic inspection specifically; Section 2.7 adapts the same value-of-information logic to the choice of which physical quantity, air temperature, emissivity, reflected radiance, distance or instrument accuracy, a field inspector should measure next.

Returning to the measurement chain itself, the atmospheric term of the measurement equation and the camera’s own radiometric stability are each treated as fixed in routine field practice but are not fixed in principle. Minkina and Klecha [56] model the infrared atmospheric transmission coefficient as a function of humidity and path length, the same physical quantity handled here by the whole-path and split-path forms of τ(d). On the camera side, Gazzano et al. [57] and Brazane et al. [58] report long-term radiometric drift in uncooled, shutterless microbolometer cameras of the kind used throughout this corpus, and Lin et al. [59] propose a pixel-wise polynomial calibration to correct spatial non-uniformity in infrared focal-plane arrays. This paper assumes a single, session-level calibration state for the camera and does not model drift or per-pixel non-uniformity separately from the measurement equation’s calibration constants, a simplification these studies suggest is not free even within a single acquisition day.

The specific measurement equation and calibration route adopted in this paper draw directly on this literature: the three-term radiometric measurement equation used throughout this paper follows the general formulation implemented in the open-source Thermimage package [60] and documented for consumer FLIR cameras by third-party reverse engineering of the file format [61]. Pušnik and Geršak [62] evaluate the size-of-source effect in thermal cameras, directly relevant to the sub-pixel boundary contamination corrected in Section 2.5, and Mendez-Bohorquez et al. [63] extend the same evaluation to a modulation-transfer-function characterisation of the effect for mid- and long-wave cameras; Dziarski et al. [64] and Hulewicz et al. [65] address thermogram sharpness and small-object measurement, respectively, as components of the same edge-spread problem; Lee and Kang [66] address the same effect from a deconvolution perspective; Ramesh et al. [67] report machine-learning calibration of smartphone-based infrared cameras, the closest recent analogue for the consumer-grade camera used in the source corpus.

The corpus used throughout this paper was deposited by Ukiwe et al. [68] as a collection of 895 raw radiometric FLIR images of five classes of substation equipment, with a companion hotspot-detection study [69]. Two 2026 papers apply an emissivity correction to a visible or auxiliary channel in a manner close enough to the present approach that they define the boundary of this paper’s contribution. Han et al. [70] segment the thermal image itself with a trained semantic-segmentation network and assign a tabulated emissivity per component, validating the correction against a single laboratory thermocouple reading on a heated insulator sample; the correction they use drops the reflected-radiance term entirely, which for a low-emissivity surface carries most of the received signal. Klingspon et al. [12] segment the camera’s own embedded visible image with an open-vocabulary model, map segments to a literature emissivity table, and solve the full measurement equation including the reflected term, evaluating on synthetic data and a high-emissivity negative control rather than a field maintenance decision. Taken together, these two papers establish visible-channel segmentation as a viable correction strategy and a high-emissivity control as a baseline sanity check; neither validates the estimated emissivity against a physical constraint that could fail, and neither propagates the resulting uncertainty to a maintenance decision. Both gaps are addressed directly in this paper.

Handheld radiometric inspection of a substation is, in this sense, a proximal (ground-based) instance of thermal remote sensing, a third observation regime alongside the satellite and the laboratory bench rather than a scaled-down version of either: a single fixed vantage point and decimetre-scale targets in place of an orbital swath or a UAV transect, but governed by the same measurement equation and open to the same correction developed here.

The contributions of the present paper are as follows.

1. In-situ emissivity estimation at material boundaries, run at corpus scale on 106 accepted metal-to-insulator junctions (91 bare-metal fittings and 15 terminals) drawn from 289 candidates across 173 frames and four equipment classes, using the boundary-continuity, reference-material principle of ASTM E1933 [71] applied retrospectively to field data rather than in a controlled laboratory setting.
2. Three tests that can reject the estimated emissivity on physical grounds without a reference thermometer: a negative control that isolates a material effect from a generic boundary artefact, a joint fit of emissivity and reflected radiance that resolves independently of the assumed reflected temperature where the data allow it, and a repeatability analysis across independent views of the same physical hardware that states the precision of the estimate rather than presenting it as exact.
3. Propagation of reflected-temperature, emissivity, air-temperature, distance, humidity and instrument-accuracy uncertainty through to the ANSI/NETA severity band for 765 graded frames, with air temperature obtained from meteorological reanalysis independently for every one of the 20 acquisition sessions rather than drawn from a climatology, together with a standardised-regression-coefficient sensitivity analysis of which input dominates the remaining uncertainty.
4. A value-of-information ranking that identifies, separately by material, which single additional measurement would most reduce the fraction of frames left without an assignable severity band, giving a field inspector without a full radiometric setup a concrete priority rather than a general recommendation to measure everything better.

## 2. Materials and Methods

### 2.1. Dataset and Acquisition Protocol

The corpus is a publicly deposited collection of 895 raw radiometric images of high-voltage substation equipment across five classes: circuit breakers, disconnectors, power transformers, surge arresters and wave traps [68]. Every image was acquired with a single consumer-grade radiometric camera (FLIR C5, 160 × 120 thermal resolution) under a single fixed set of acquisition parameters, applied uniformly regardless of session, equipment class or time of day: emissivity ε = 0.95, object distance 10 m, reflected and atmospheric temperature 20.0 °C, and relative humidity 50%. Each raw file additionally carries a co-registered visible-light photograph at 640 × 480 resolution, embedded by the camera as a documentation aid, and a burnt-in colour-scale readout showing the value at whichever measurement tool, a spot or an area box, the operator had active at the moment of capture. Two files (FLIR1076, FLIR1080) have corrupted image data and are excluded, leaving 893 usable frames. The 893 frames resolve into 20 distinct acquisition sessions by inspection of embedded timestamps and equipment continuity; 18 of the 20 sessions each hold a single equipment class.

**Figure 1:**
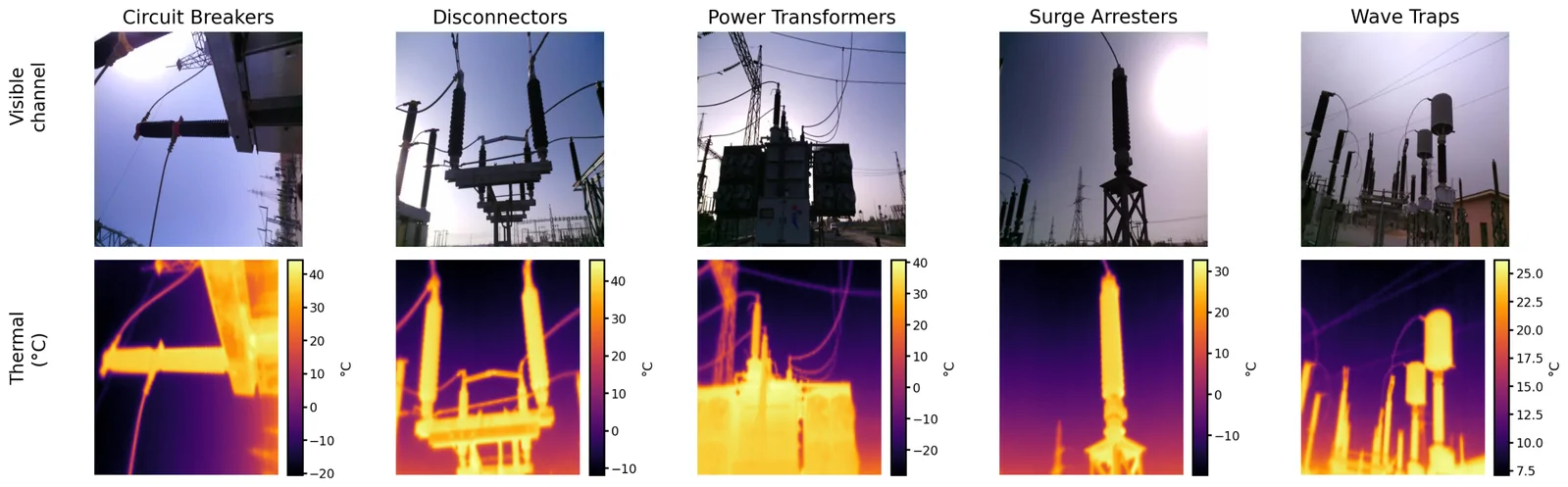
Representative visible and thermal frames, one per equipment class. Top row: the embedded visible-light photograph. Bottom row: the corresponding radiometric thermal frame, rendered in pseudocolour with its own temperature scale.

**Figure 2:**
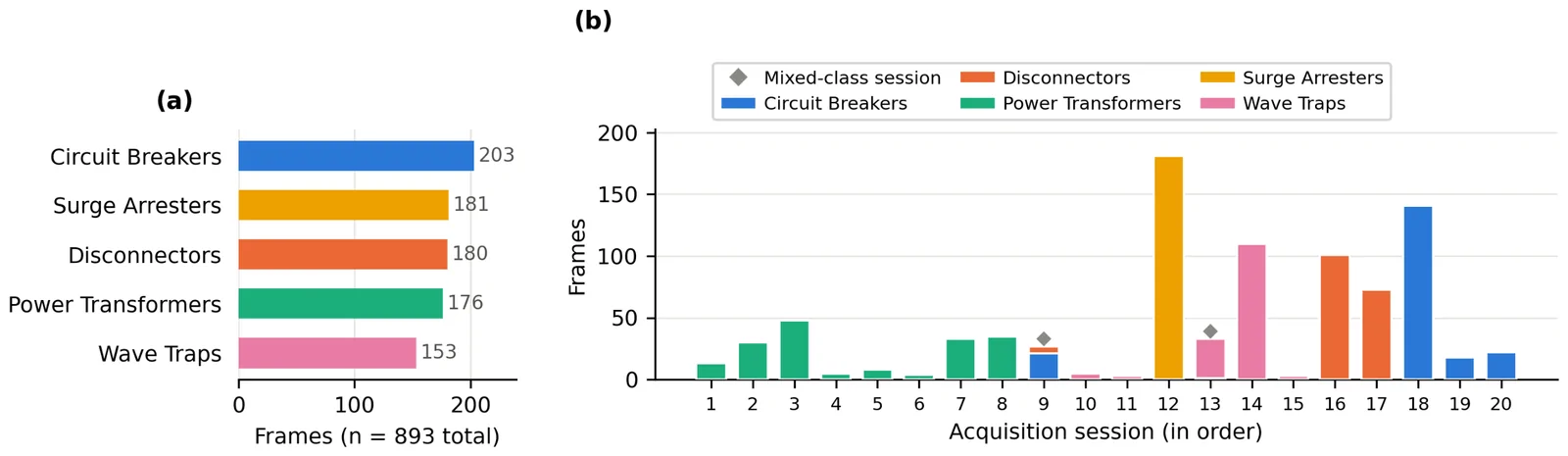
Corpus composition. (**a**) Frames per equipment class. (**b**) Frames per acquisition session, coloured by equipment class.

Figure 1 shows a representative visible and thermal frame pair for each equipment class, illustrating both the field of view returned by the visible channel and the wide range of apparent temperature and scene content the fixed acquisition settings are applied to uniformly. Figure 2 summarises the resulting corpus composition, both by equipment class and by acquisition session; the concentration of most sessions on a single equipment class (Figure 2b) is what later restricts the boundary validation of Section 2.6 to a subset of classes (Section 4.3).

### 2.2. Radiometric Measurement Equation and Chain Correction

The camera’s internal calibration relates a corrected digital signal *S* to an apparent temperature *T* through a Planck-derived function of the form

(1) $$S\left(T\right)=\frac{R_{1}}{R_{2}\left(e^{B/T}-F\right)}-O$$

where *T* is in kelvin and $R_{1}$, $R_{2}$, *B*, *F* and *O* are calibration constants specific to the detector and lens combination. The total signal received by the detector decomposes into three physical sources, following the standard measurement equation for radiometric thermal cameras [4,60]:

(2) $$S_{\mathrm{tot}}=\varepsilon \;\tau \left(d\right)\;S\left(T_{\mathrm{obj}}\right)+\left(1-\varepsilon \right)\;\tau \left(d\right)\;S\left(T_{\mathrm{refl}}\right)+\left[1-\tau (d)\right]S\left(T_{\mathrm{atm}}\right)$$

where τ(d) is the atmospheric transmittance over the object distance d, $T_{\mathrm{refl}}$ is the effective temperature of radiation reflected off the object surface, and $T_{\mathrm{atm}}$ is the temperature of the intervening atmosphere. Inverting Equation (2) for the object signal and Equation (1) for the corresponding temperature gives the recovered object temperature   

(3) $$\begin{array}{c} T_{\mathrm{obj}}=\frac{B}{\ln\;\left\{\frac{R_{1}}{R_{2}\left(S_{\mathrm{obj}}+O\right)}+F\right\}},\\ S_{\mathrm{obj}}=\frac{S_{\mathrm{tot}}-\left(1-\varepsilon \right)\;\tau \left(d\right)\;S\left(T_{\mathrm{refl}}\right)-\left[1-\tau (d)\right]S\left(T_{\mathrm{atm}}\right)}{\varepsilon \;\tau (d)} \end{array}$$

Two implementations of τ(d) appear in the literature and disagree on how the object distance enters: a whole-path form that evaluates the transmittance once over the full distance d, and a split-path form, used in the open-source Thermimage package [60], that evaluates it over half the distance and squares the result to represent a round trip:

(4) $$\tau_{\mathrm{split}}\left(d\right)={\left[\tau (d/2)\right]}^{2}$$

**Figure 3:**
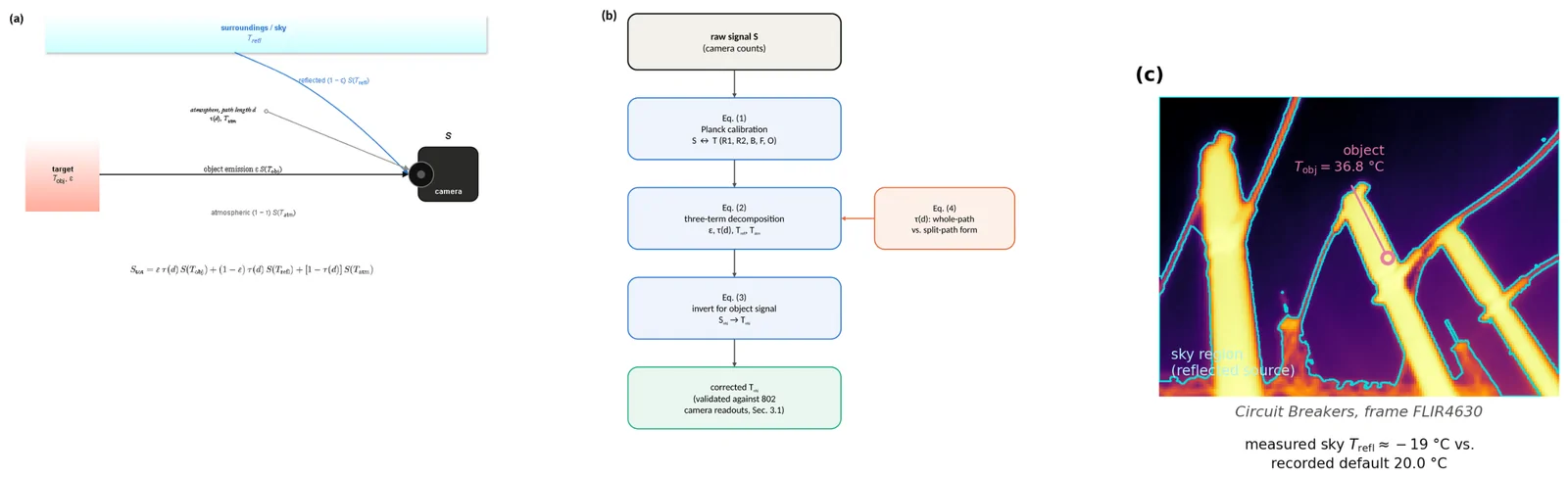
The radiometric measurement equation and its correction chain. (**a**) Radiative-transfer decomposition of the signal received by the camera into object, reflected and atmospheric contributions (Equation (2)). (**b**) Correction pipeline from the raw signal to the corrected object temperature, showing where each of Equations (1) to (4) enters. (**c**) The same terms located on a representative acquired frame (circuit breaker, session 20): the graded object point and the segmented sky region that supplies the reflected term, with the measured sky temperature contrasted against the camera’s recorded default.

Figure 3 summarises this correction chain: panel (a) shows the three-term radiative decomposition of Equation (2), panel (b) traces the pipeline from the raw signal through Equations (1) to (4) to the corrected object temperature validated in Section 3.1, and panel (c) locates the same three terms on one acquired frame, where the measured sky (reflected) temperature departs from the recorded default by close to 40 K.

793 of the 893 usable frames carry a burnt-in readout, printed by the camera itself to 0.1 K precision, at the position of its active measurement tool (area box in 421 files, spot in 362, both in 10). These 802 plain readouts (11 further readouts are flagged by the camera as out of range and are treated separately) give an independent check on which recovery is correct, because the readout is the camera’s own solution of Equation (3) under its own recorded parameters, decoded here from the printed digits with a template-based reader verified by eye on a random sample of 80 readouts (all 80 correct). Comparing the whole-path recovery, the split-path recovery, and a third, direct-inversion recovery that inverts the calibration without re-deriving the atmospheric term against these readouts identifies which transmittance model the camera itself uses, and validates the corrected chain used for every subsequent step in this paper.

### 2.3. Visible-Channel Registration and Scene Radiance Estimation

The embedded photograph and the thermal frame are related by a fixed scale factor, given by the camera’s stored Real2IR parameter, and a translation that the camera also stores (OffsetX, OffsetY), but that does not in practice match the true alignment. The translation is instead estimated per frame by maximising the correlation between gradient magnitudes of the photograph, downsampled to the thermal grid, and the thermal field itself,

(5) $$\left(\Delta x^{*},\;\Delta y^{*}\right)=\underset{\Delta x,\;\Delta y}{\mathrm{arg max}}\;\rho \;\left(\left|\nabla P_{↓}\left(x-\Delta x,\;y-\Delta y\right)\right|,\;\left|\nabla T(x,y)\right|\right)$$

where $P_{↓}$ is the photograph resampled onto the thermal grid, ∇ denotes the image gradient magnitude, and ρ is the Pearson correlation coefficient evaluated over the overlapping field of view for a candidate translation (Δx, Δy). A confident registration (correlation peak ρ* ≥ 0.35 and at least 1.1 times the second-highest peak) was obtained for 831 of the 893 frames with intact photographs. This procedure estimates the translation independently for every frame from image content and does not use the camera’s own embedded, distance-dependent parallax correction, which is generally a single, session-level translation and is not expected to be accurate at the standoff distances used in this corpus; the corpus’s own recorded object distance (10 m, fixed for every frame, Section 2.1) is correspondingly not used anywhere in the registration or in the radiometric correction chain, which instead treats distance as an uncertain nuisance parameter (Section 2.7).

Once registered, the photograph is classified pixel-by-pixel into sky and non-sky regions using HSV colour (saturated blue, or bright neutral within 30 K of the blue-sky thermal reading), a local texture test exploiting the visual smoothness of clear sky, and removal of small connected components. The thermal signal within the resulting sky mask $M_{\mathrm{sky}}$ gives a per-frame apparent sky temperature,

(6) $$T_{\mathrm{refl}}=\underset{\left(x,y\right)\;\in \;M_{\mathrm{sky}}}{\mathrm{median}}\;T\left(x,y\right)$$

one of the two physical sources of reflected radiance at an outdoor substation (the other being warm structure and equipment elsewhere in the field of view). This measured sky temperature replaces the fixed 20.0 °C reflected-temperature assumption everywhere in this paper except where explicitly noted otherwise.

**Figure 4:**
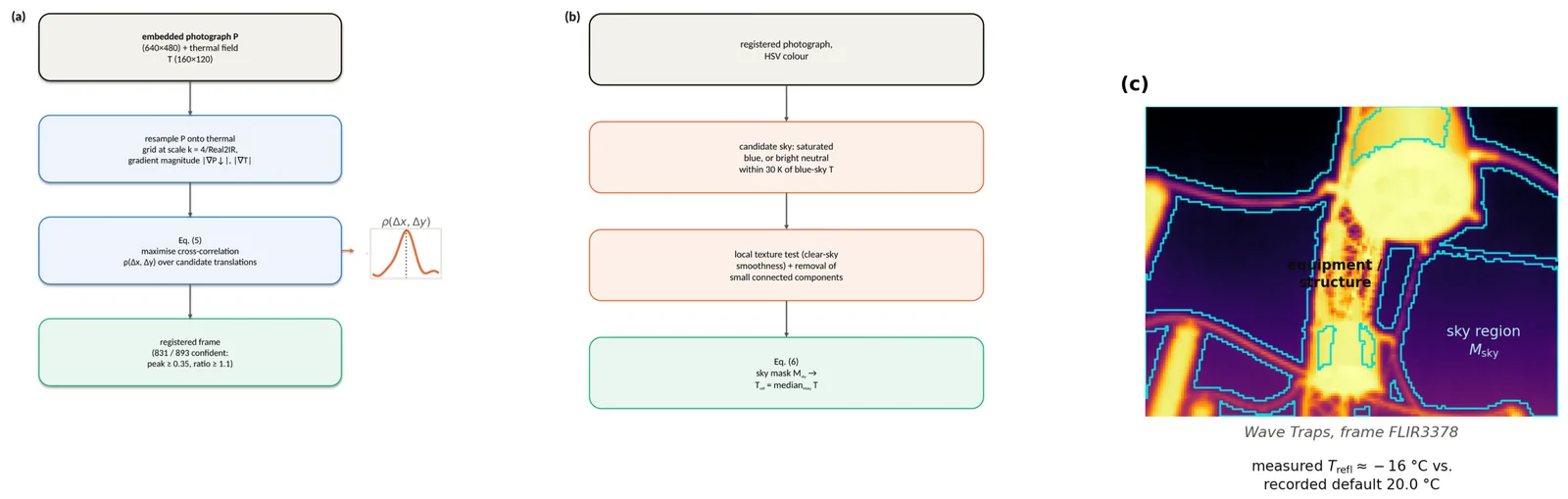
Visible-channel registration and sky-radiance estimation. (**a**) Per-frame registration pipeline, maximising the gradient correlation between the resampled photograph and the thermal field (Equation (5)). (**b**) Sky-classification pipeline from the registered photograph to the sky mask and its apparent temperature (Equation (6)). (**c**) The recovered sky mask and measured sky (reflected) temperature on one representative acquired frame (wave trap, session 14), contrasted with the camera’s recorded default.

Figure 4 summarises this section: panel (a) traces the per-frame registration pipeline that maximises the gradient correlation of Equation (5), panel (b) traces the sky-classification pipeline feeding the sky-temperature statistic of Equation (6), and panel (c) locates the recovered sky mask on one acquired frame, where the measured sky temperature departs from the recorded default by more than 35 K.

### 2.4. Material Labelling at the Graded Location

Severity grading and emissivity estimation both require knowing what surface is at the location a frame actually grades. This graded location is the position of the camera’s active measurement tool when one is stored, or, when no tool is stored, the centroid of the hottest non-sky region in the frame, taken as the non-sky pixels at or above the 99.5th percentile of the frame’s temperature distribution:

(7) $$p^{*}=\left\{\begin{array}{cc} p_{\mathrm{tool}}, & \mathrm{measurement}\;\mathrm{tool}\;\mathrm{stored}\\ \mathrm{centroid}\;\left\{\left(x,y\right)\notin M_{\mathrm{sky}}:T\left(x,y\right)\geq T_{99.5}\right\}, & \mathrm{otherwise} \end{array}\right.$$

Each of the 893 graded locations was assigned one of seven material codes by a human reviewer from a paired photograph and thermal crop: insulator housing (glazed porcelain or silicone rubber), painted surface, bare or lightly oxidised metal, galvanised steel, sheet-metal housing, background (not on equipment), or not determinable. This is a single point label per frame, not a per-pixel segmentation of the scene; the local, geometric segmentation used to find material boundaries for emissivity estimation (Section 2.6) is a separate, narrower procedure restricted to a small window around this same point.

**Figure 5:**
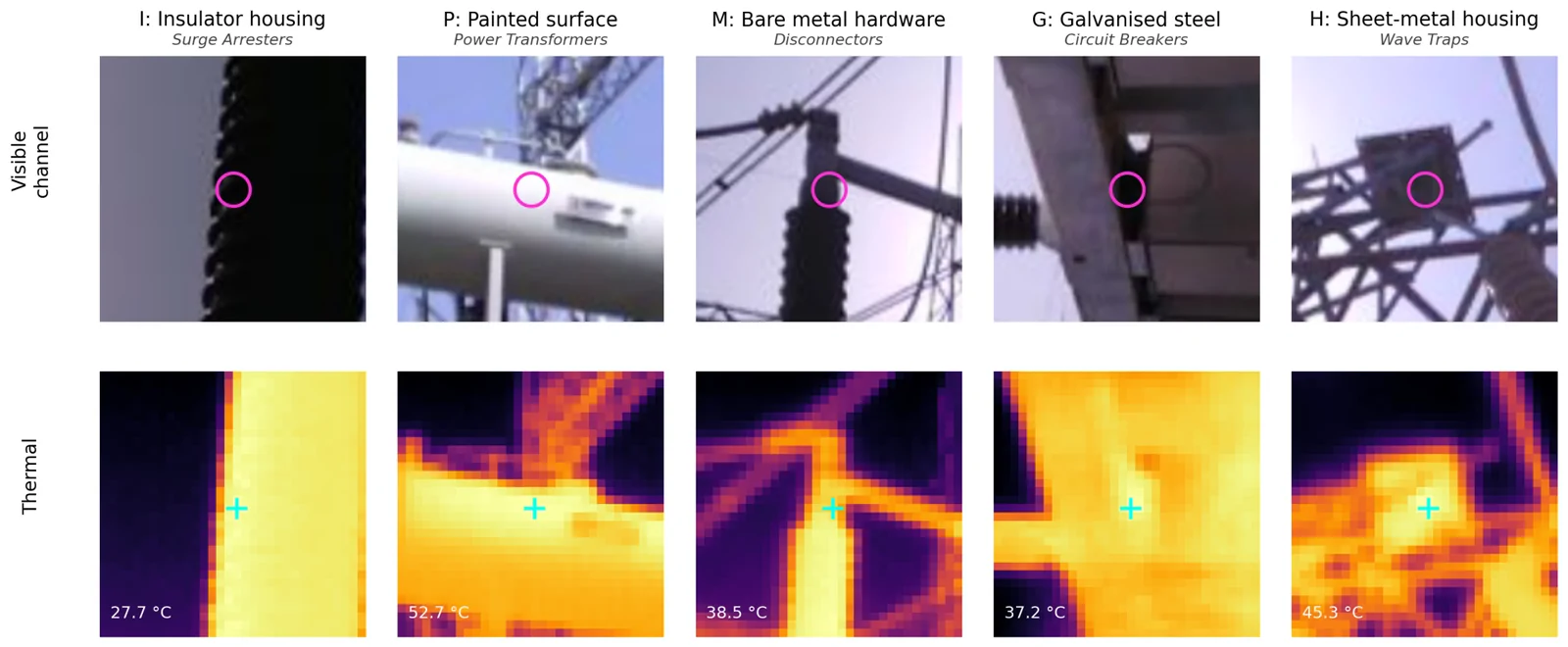
Material labelling at the graded point (marked), one representative frame per material code, each drawn from a different equipment class (named beneath the material code). Top row: the registered visible crop the reviewer labelled from, with the graded point marked by a magenta circle. Bottom row: the corresponding thermal crop, with the same point marked by a cyan cross and its recovered temperature given beneath.

Figure 5 shows this labelling procedure directly: the registered visible crop and the corresponding thermal crop around the graded point (marked), for one representative frame of each of the five material codes that carry a distinct emissivity prior in Section 2.7. Because every material code occurs on more than one equipment class in the corpus, the five examples are drawn from five different equipment classes rather than repeating one, so the figure illustrates the labelling procedure across the full range of equipment, not a single class. The visible crop is what the reviewer actually labelled from; the thermal crop is shown only for context and played no part in the material decision, which prevents the temperature at a location from biasing the label assigned to it.

### 2.5. Point-Spread Correction for Boundary Contamination

A pixel located close to a sky-to-object boundary receives radiance mixed from both sides of the boundary to the extent that the camera’s thermal point-spread function straddles the edge, which biases any measurement taken close to a material boundary, including both the junction cores used for emissivity estimation (Section 2.6) and the reference surfaces used for the relative severity criterion (Section 2.7). The signal profile across such a boundary, along the coordinate x normal to the edge, is modelled as an error-function transition between the sky and object plateaus,

(8) $$S\left(x\right)=S_{\mathrm{sky}}+\frac{S_{\mathrm{obj}}-S_{\mathrm{sky}}}{2}\left[1+\mathrm{erf}\;\left(\frac{x-x_{0}}{\sigma \sqrt{2}}\right)\right]$$

where $x_{0}$ is the edge position and σ is the point-spread sigma. The corresponding fractional contamination of a pixel centred a distance x inside the object side is

(9) $$c\left(x\right)=\frac{1}{2}\;\mathrm{erfc}\;\left(\frac{x}{\sigma \sqrt{2}}\right)$$

The sigma was fitted from 32,963 sampled sky-to-object edges across all 20 sessions and used to set a minimum clearance, in pixels, that both the junction cores of Section 2.6 and the reference surfaces of Section 2.7 must respect from any sky boundary.

**Figure 6:**
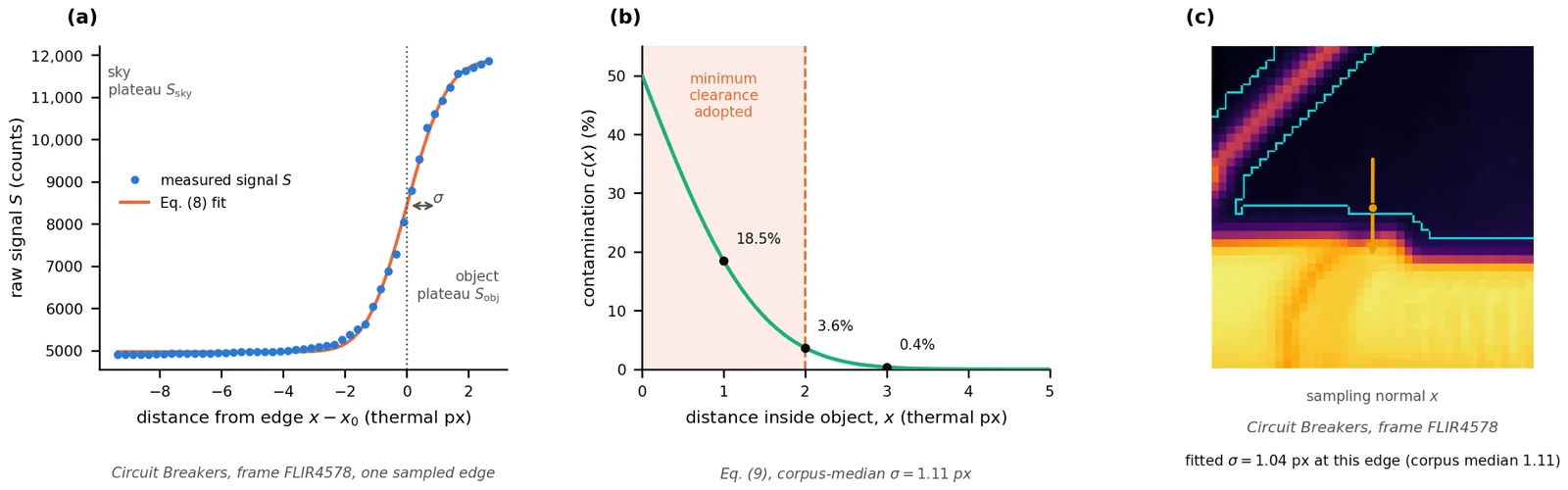
Point-spread correction on one sampled edge. (**a**) Raw signal profile across a sky-to-object edge and its error-function fit (Equation (8)). (**b**) Fractional contamination as a function of distance inside the object (Equation (9)), evaluated at the corpus-median point-spread sigma, with the adopted minimum clearance shaded. (**c**) The sampled edge and its normal direction located on the acquired frame (circuit breaker, session 19).

Figure 6 illustrates this correction on one sampled edge: panel (a) shows the raw signal profile and its erf fit of Equation (8), panel (b) shows the resulting contamination curve of Equation (9) and the clearance it sets, and panel (c) locates the sampled edge and its normal direction on the acquired frame.

### 2.6. In-Situ Emissivity Estimation at Material Boundaries

**Candidate junctions.** Around each graded location classified as insulator or metal, a local, geometric (non-learned) segmentation of the registered photograph into dark (housing or insulator) and mid-grey, non-blue (bare metal) connected components identifies adjacent regions in direct contact. From each pair, two “cores”, rectangles two to six pixels inside the boundary on either side and clear of sky contamination beyond the tolerance set in Section 2.5, give a metal signal $S_{m}$ and an anchor (insulator) signal $S_{a}$. This procedure yielded 289 candidate junctions across 173 frames, each subsequently reviewed by eye against a paired photo/thermal sheet and coded as an accepted bare-metal fitting, an accepted terminal or conductor, an undetermined housing, a painted surface, or rejected.

**Closed-form emissivity at an anchored junction.** Two surfaces in direct thermal contact share a junction temperature $T_{j}$ at the interface. If the anchor’s emissivity $\varepsilon_{a}$ is known from an established reference value (glazed porcelain, $\varepsilon_{a}$ = 0.92), its own recovered object temperature via Equation (3) gives $T_{j}$, and the raw signals $S_{m}$ and $S_{a}$ of the two cores under a common reflected temperature $T_{\mathrm{refl}}$ and transmittance τ give the metal’s emissivity in closed form. Writing S(T) for the Planck-type response of Equation (1), Equation (2) applied separately to the metal and the anchor and subtracted gives

$$S_{m}-S_{a}=\tau \left[S\left(T_{j}\right)-S\left(T_{\mathrm{refl}}\right)\right]\left(\varepsilon_{m}-\varepsilon_{a}\right)$$

so that

(10) $$\varepsilon_{m}=\varepsilon_{a}+\frac{S_{m}-S_{a}}{\tau \left[S\left(T_{j}\right)-S\left(T_{\mathrm{refl}}\right)\right]}$$

This closed form requires an assumed value for $T_{\mathrm{refl}}$; three values are used throughout, the recorded 20.0 °C, the frame’s own measured sky temperature (Section 2.3), and a warmer-surround bracket of sky temperature plus 10 K.

**Sensitivity to an unmodelled temperature gradient between the two cores.** Equation (10) assumes the metal and anchor cores share one junction temperature $T_{j}$. Different thermal conductivities, convective exchange and contact resistance between the two materials can instead leave a small, unmodelled gradient δ between them (Section 4.2 of Sun et al. [72] reviews the physical scale of such gradients at conforming metal-to-non-metal contacts). Taylor-expanding Equation (10) to first order in δ (the metal core truly at $T_{j}+\delta$, the anchor still at $T_{j}$) gives the resulting bias in the recovered emissivity,

$$\varepsilon_{m,\mathrm{recovered}}-\varepsilon_{m,\mathrm{true}}\;\approx \;\varepsilon_{m,\mathrm{true}}\;\delta \;\frac{S^{\prime }\left(T_{j}\right)}{S\left(T_{j}\right)-S\left(T_{\mathrm{refl}}\right)}$$

where $S^{\prime }\left(T\right)$ is the slope of the Planck-type response of Equation (1): the bias scales as $1/[S\left(T_{j}\right)-S\left(T_{\mathrm{refl}}\right)]$ and diverges as the junction temperature approaches the assumed reflected temperature, a badly conditioned regime also noted for underdetermined radiometric inverse problems generally [73]. For all 106 accepted junctions, using only the already-reported closed-form values of Section 3.6, $|T_{j}-T_{\mathrm{refl}}|$ ranges from 35.5 to 78.8 K (median 45.7 K); forward-modelling the metal core’s signal at $T_{j}+\delta$ and recovering $\varepsilon_{m}$ with the unmodified estimator gives a median bias of 0.011 at δ=0.5 K, 0.022 at δ=1 K, 0.045 at δ=2 K and 0.114 at δ=5 K (worst case throughout at the junction closest to this regime, $|T_{j}-T_{\mathrm{refl}}|=35.5$ K: 0.015, 0.031, 0.062 and 0.157 respectively). No accepted junction in this corpus is close enough to the degenerate regime for this bias to be material at a plausible gradient size.

**Joint estimation of the emissivity ratio and reflected radiance.** Where several junctions within a session share a metal type and a common reflected radiance, the reflected temperature need not be assumed at all. Writing $B_{\mathrm{atm}}=S\left(T_{\mathrm{atm}}\right)$ for the atmospheric path radiance and correcting the anchor and metal signals individually for the atmospheric term before fitting, $x=(S_{a}-\left(1-\tau \right)B_{\mathrm{atm}})/\tau$ and $y=(S_{m}-\left(1-\tau \right)B_{\mathrm{atm}})/\tau$, the same two measurement equations give a relation linear in *x*:

(11) $$y=k\;x+\left(1-k\right)\;S\left(T_{\mathrm{refl}}\right),\;k\equiv \frac{\varepsilon_{m}}{\varepsilon_{a}}$$

so that a robust linear regression (Theil-Sen, bootstrapped for confidence intervals) of *y* on *x* across junctions of one session and equipment class gives the emissivity ratio k directly from the slope, independent of any assumed reflected temperature, and the reflected temperature itself from the intercept c of that fit,

(12) $$T_{\mathrm{refl}}=S^{-1}\;\left\{\frac{c}{1-\hat{k}}\right\}$$

where $S^{-1}$ denotes Equation (1) solved for T. Restated for the raw recorded signals rather than the atmosphere-corrected *x* and *y*, Equation (11) is $S_{m}=k\;S_{a}+\left(1-k\right)\left[\tau \;S\left(T_{\mathrm{refl}}\right)+\left(1-\tau \right)\;B_{\mathrm{atm}}\right]$; fitting the atmosphere-corrected quantities directly, as done here, is algebraically equivalent to this raw-signal form but removes the additional assumption that τ and $T_{\mathrm{atm}}$ are constant across the pooled junctions, since each junction is corrected individually with its own value of both before the regression is run. This joint fit was attempted on the four sessions with five or more accepted junctions, and additionally, for the three sessions, sharing an equipment class but individually too narrow in temperature spread to constrain the slope, as a fixed-effects fit with a slope shared across the three sessions and a separate reflected radiance per session.

**Validation without a reference thermometer.** Because this is a retrospective public corpus, no contact reference temperature exists at any junction, so three tests validate the estimate on physical grounds instead. First, a within-insulator negative control repeats the same core-extraction procedure between two points on the same insulator housing, 8 to 12 pixels apart, straddling no material boundary; if the metal-versus-insulator step were a generic near-boundary artefact rather than a material effect, this pseudo-step should show the same pattern, tested with a Wilcoxon signed-rank test against the real step. Second, the joint fit of Equation (11) is cross-checked by a drift scan: scanning the assumed reflected temperature and locating where the Spearman correlation between implied emissivity and junction temperature crosses zero, which an unconfounded reflected-temperature estimate should also identify, independently of the regression. Third, repeatability across independent views of the same physical hardware is quantified with a one-way random-effects intraclass correlation, ICC(1), computed over “components,” consecutive accepted junctions within four frames of each other and with the metal core centroid moving less than 15 pixels, following

(13) $$\mathrm{ICC}\left(1\right)=\frac{MS_{B}-MS_{W}}{MS_{B}+\left(k_{0}-1\right)\;MS_{W}}$$

where MSB and MSW are the between- and within-component mean squares from a one-way analysis of variance and $k_{0}$ is the standard correction for unequal group sizes [74]. Algorithm 1 states the corpus-scale procedure end-to-end, from candidate-junction extraction through the closed-form and joint emissivity routes to the three reference-free validation tests.

**Figure 7:**
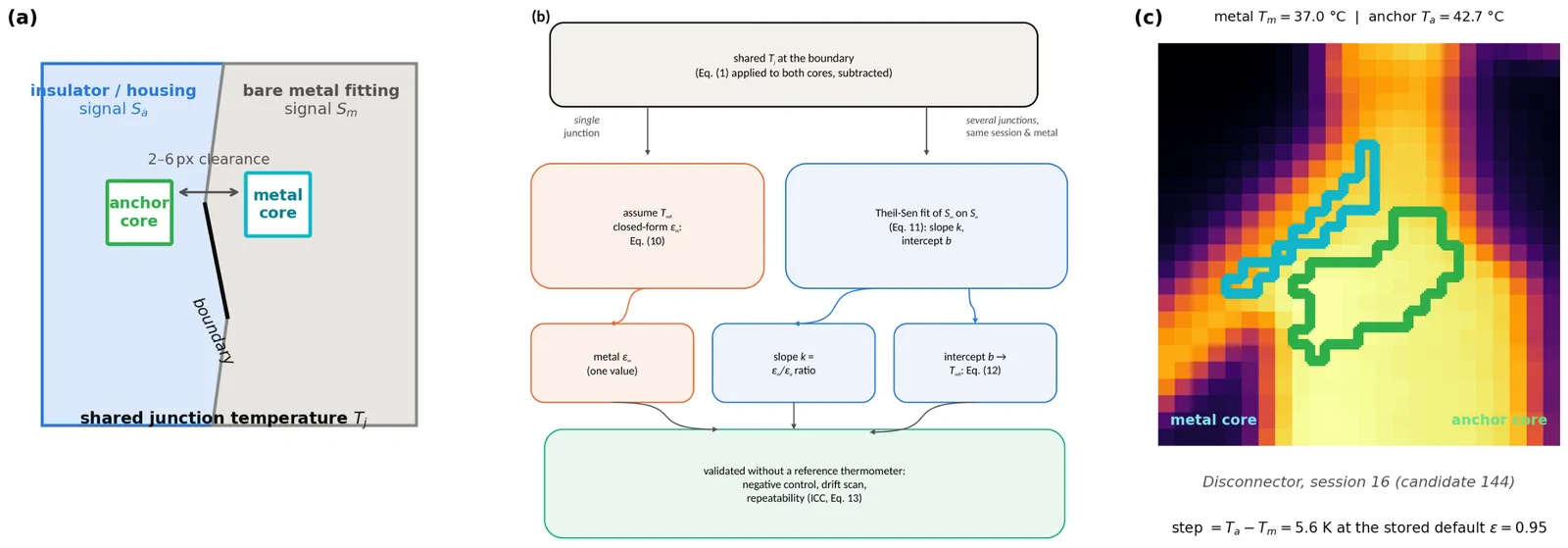
In-situ emissivity estimation at material boundaries. (**a**) Geometry of a candidate junction: anchor and metal cores positioned two to six pixels either side of the segmented boundary, sharing a junction temperature. (**b**) The two estimation routes, a single-junction closed form (Equation (10)) and a joint multi-junction fit (Equations (11) and (12)), both validated without a reference thermometer (Equation (13)). (**c**) The two cores located on one representative accepted junction (disconnector, session 16): the anchor and metal temperatures recovered at the stored default emissivity, and their difference.

Figure 7 summarises this section: panel (a) shows the junction geometry, the anchor and metal cores on either side of the boundary, panel (b) contrasts the single-junction closed-form route of Equation (10) with the joint multi-junction fit of Equations (11) and (12), both converging on the reference-free validation of Equation (13), and panel (c) locates both cores on one accepted junction, where the two read 5.6 K apart at the stored default emissivity.
**Algorithm 1** Corpus-scale in-situ emissivity estimation at metal-to-insulator junctions**Require:** registered photograph *P*, thermal field *T* (Section 2.3), sky-contamination clearance (Section 2.5)**Ensure:** accepted junctions with recovered metal emissivity, and session reflected temperature where jointly estimable  1:J←∅  2:for each frame with a graded location labelled insulator or metal do  3:    segment the registered photograph into dark (housing or insulator) and mid-grey, non-blue (bare-metal) connected components  4:      for each pair of adjacent dark and metal components in direct contact do  5:            place an anchor core and a metal core, 2 to 6 pixels inside the boundary, clear of sky contamination (Section 2.5)  6:            $S_{a},S_{m}\leftarrow$ mean raw signal of the anchor core, the metal core  7:            $J\leftarrow J\cup \{\left(S_{a},S_{m},\mathrm{frame},\mathrm{session},\mathrm{equipment}\;\mathrm{class}\right)\}$  8:      end for  9:end for                                     ▹|J|=289 candidate junctions across 173 frames10:review every junction in *J* against a paired photo/thermal sheet; retain the accepted bare-metal fittings and terminals                                                    ▹ 106 of 289 accepted11:for each accepted junction do                                      ▹ single-junction route12:      $T_{j}\leftarrow S^{-1}\left(S_{a};\varepsilon_{a}=0.92,T_{\mathrm{refl}}\right)$                                        ▹ Equation (3)13:      $\varepsilon_{m}\leftarrow$ closed form of Equation (10) from $S_{m}$, $S_{a}$, $T_{\mathrm{refl}}$ and τ14:end for15:for each session and equipment class with 5 or more accepted junctions do                      ▹ joint route16:      k,c← Theil-Sen fit of $y=(S_{m}-\left(1-\tau \right)B_{\mathrm{atm}})/\tau$ on $x=(S_{a}-\left(1-\tau \right)B_{\mathrm{atm}})/\tau$, bootstrapped for confidence intervals                                                        ▹ Equation (11)17:      $T_{\mathrm{refl}}\leftarrow S^{-1}\left(c/\left(1-k\right)\right)$                                          ▹ Equation (12)18:end for19:validate $\varepsilon_{m}$: within-insulator negative control, reflected-temperature drift scan on the joint fit, ICC(1) across independent views                                                       ▹ Equation (13)20:return the accepted junctions with $\varepsilon_{m}$, $T_{j}$ and the validation statistics

### 2.7. Uncertainty Propagation to Severity Bands

For each of the 765 frames graded insulator, painted, bare metal, galvanised or sheet-metal housing, a per-frame Monte Carlo of N = 3000 draws propagates the dominant sources of uncertainty through Equation (3) to the temperature rise that ANSI/NETA MTS Table 100.18 [1] actually grades. Two rises are computed per draw, one against the ambient air temperature and one against a photometrically similar reference surface found elsewhere in the same frame:

(14) $$\Delta T_{\mathrm{air}}=T_{\mathrm{obj}}-T_{\mathrm{air}},\;\Delta T_{\mathrm{rel}}=T_{\mathrm{obj}}-T_{\mathrm{ref}}$$

The Monte Carlo inputs are: surface emissivity, drawn from a material-dependent prior informed directly by the corpus-scale junction results of Section 2.6 (for example, 0.90 to 0.95 for insulator housing and 0.45 to 0.90 for bare metal); reflected radiance, a mixture of the frame’s measured sky temperature (Section 2.3) and a warmer surrounding-structure temperature; air temperature, drawn around a value obtained independently for every one of the 20 sessions from meteorological reanalysis, as the mean of two hourly reanalyses (Open-Meteo and NASA POWER) over each session’s capture window, bracketed by the larger of 1.5 K and half the two sources’ disagreement, which bounds disagreement between the two sources at the assumed location rather than uncertainty in the location itself (Section 3.10); object distance, U(3, 15) m; relative humidity, U(0.15, 0.90); and the camera’s own stated instrument accuracy, ±3 K, applied to the ambient criterion only. Each of the two rises is mapped, per draw, to one of the four ANSI/NETA bands (thresholds 1, 10, 20 and 40 K for the ambient criterion; 1, 3 and 15 K for the relative criterion), giving a modal band and its empirical probability across the 3000 draws for every frame.

Sensitivity of the rise to each input is summarised by the standardised-regression-coefficient-squared (SRC^2^) of that input, computed per frame by an ordinary-least-squares fit of the rise on the standardised Monte Carlo draws:

(15) $${\mathrm{SRC}}_{i}^{2}=\beta_{i}^{2},\;\sum_{i}{\mathrm{SRC}}_{i}^{2}\approx R^{2}$$

where $\beta_{i}$ is the regression coefficient of input i on the standardised output and R^2^ is the coefficient of determination of the linear surrogate. A value-of-information analysis starts from the baseline above and progressively narrows the remaining unknowns in four nested scenarios, air temperature known to ±0.5 K, instrument error removed, reflected radiance known, and finally emissivity known to ±0.05 at worst, each re-run as a completely separate Monte Carlo to identify which single additional measurement most reduces the fraction of frames left without an assignable band. Algorithm 2 states the per-frame propagation and the nested value-of-information ranking together.

**Algorithm 2** Per-frame Monte Carlo uncertainty propagation and value-of-information ranking
**Require:** graded frame with material label, corrected measurement equation (Equation (3)), material-dependent emissivity prior, per-frame sky temperature (Section 2.3), session air temperature, N=3000**Ensure:** modal ANSI/NETA band and confidence under both grading criteria; input sensitivity; value-of-information ranking by material
  1:for each of the 765 graded frames do  2:      for n=1 to *N* do  3:          draw ε from the material prior; $T_{\mathrm{refl}}$ from the sky and structure mixture; $T_{\mathrm{air}}$ from the session estimate; *d*, U(3,15) m; RH, U(0.15,0.90); instrument bias, ±3 K  4:          $\Delta T_{\mathrm{ambient}},\Delta T_{\mathrm{reference}}\leftarrow$ temperature rise via Equations (3) and (14), against air temperature and against the in-frame reference surface  5:          ${\mathrm{band}}_{\mathrm{ambient}}\leftarrow$ ANSI/NETA band of $\Delta T_{\mathrm{ambient}}$ (thresholds 1, 10, 20, 40 K)  6:          ${\mathrm{band}}_{\mathrm{reference}}\leftarrow$ ANSI/NETA band of $\Delta T_{\mathrm{reference}}$ (thresholds 1, 3, 15 K)  7:      end for  8:      modal band, confidence ← mode and empirical probability of ${\mathrm{band}}_{\mathrm{ambient}}$ and of ${\mathrm{band}}_{\mathrm{reference}}$ over the *N* draws  9:      compute the standardised-regression-coefficient-squared of each input on the rise ▹ Equation (15)10:end for11:for each of four nested scenarios (air temperature to ±0.5 K; instrument error removed; reflected radiance known; emissivity known to ±0.05) do12:      re-run lines 2 to 9 with that input fixed13:      record the change in the fraction of frames left without an assignable band, by material14:end for15:return the per-frame modal bands and confidence, the sensitivity ranking, and the value-of-information ranking by material


## 3. Experimental Evaluation

### 3.1. Radiometric Chain Validation Against Camera Readouts

**Figure 8:**
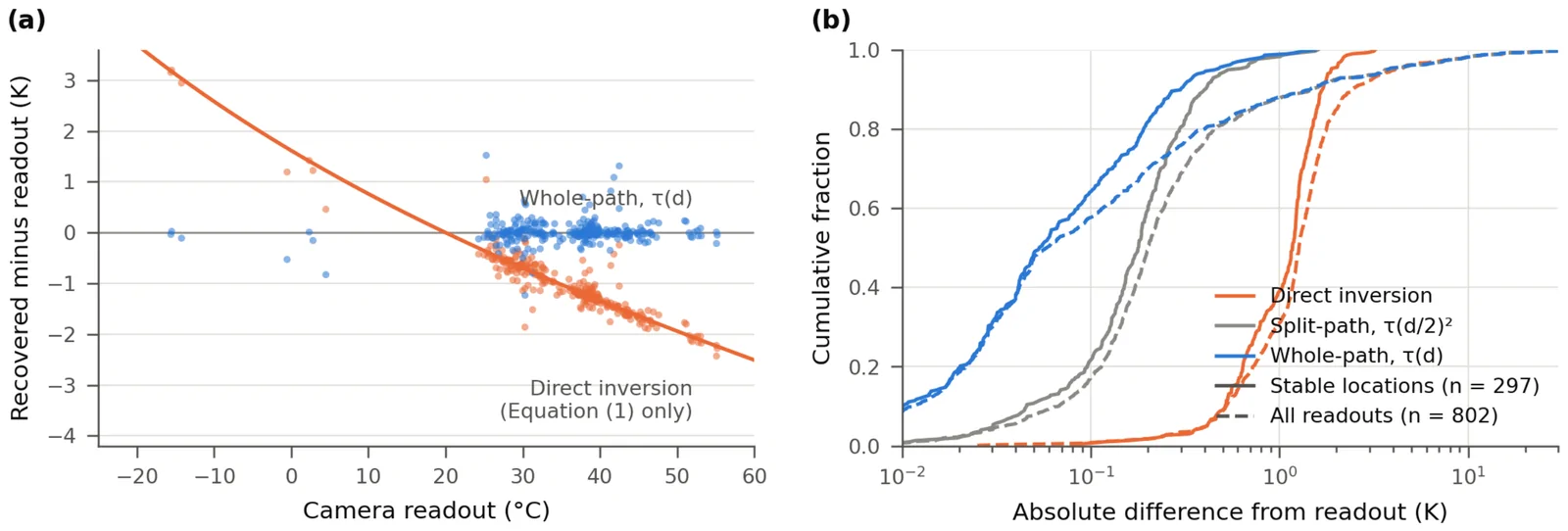
Radiometric chain validation. (**a**) Recovered-minus-readout difference against readout value for the whole-path and direct-inversion recoveries. (**b**) Cumulative distribution of the absolute difference for all three recoveries, stable and full readout sets.

Table 1 compares the three candidate recoveries of Section 2.2 against the 802 plain camera readouts. The whole-path measurement equation matches the camera’s own readouts far more closely than either alternative, with a median difference of −0.01 K against −1.17 K for the direct-inversion recovery that omits the atmospheric re-derivation and +0.15 K for the split-path form used in Thermimage [60]. Fitting the transmittance freely against the 297 most stable readouts (a spot whose 3 × 3 neighbourhood spans under 1 K, or a box extreme at least three pixels inside the box) gives τ = 0.9763, matching the camera’s own stored whole-path coefficient (0.9761) rather than its split-path equivalent (0.9664), which confirms that the camera itself implements the whole-path form. Figure 8 shows the signed difference against the readout value (panel a) and the cumulative distribution of the absolute difference for all three recoveries (panel b), separately for all 802 readouts and for the 297 stable subset. No acquisition session carries a systematic offset (session medians between −0.22 and +0.05 K), and the largest residual differences concentrate at steep local gradients and box edges rather than in any particular session, consistent with a small remaining spatial registration error between the printed readout and the stored frame rather than a radiometric bias.

### 3.2. Registration Accuracy and Scene Radiance

**Figure 9:**
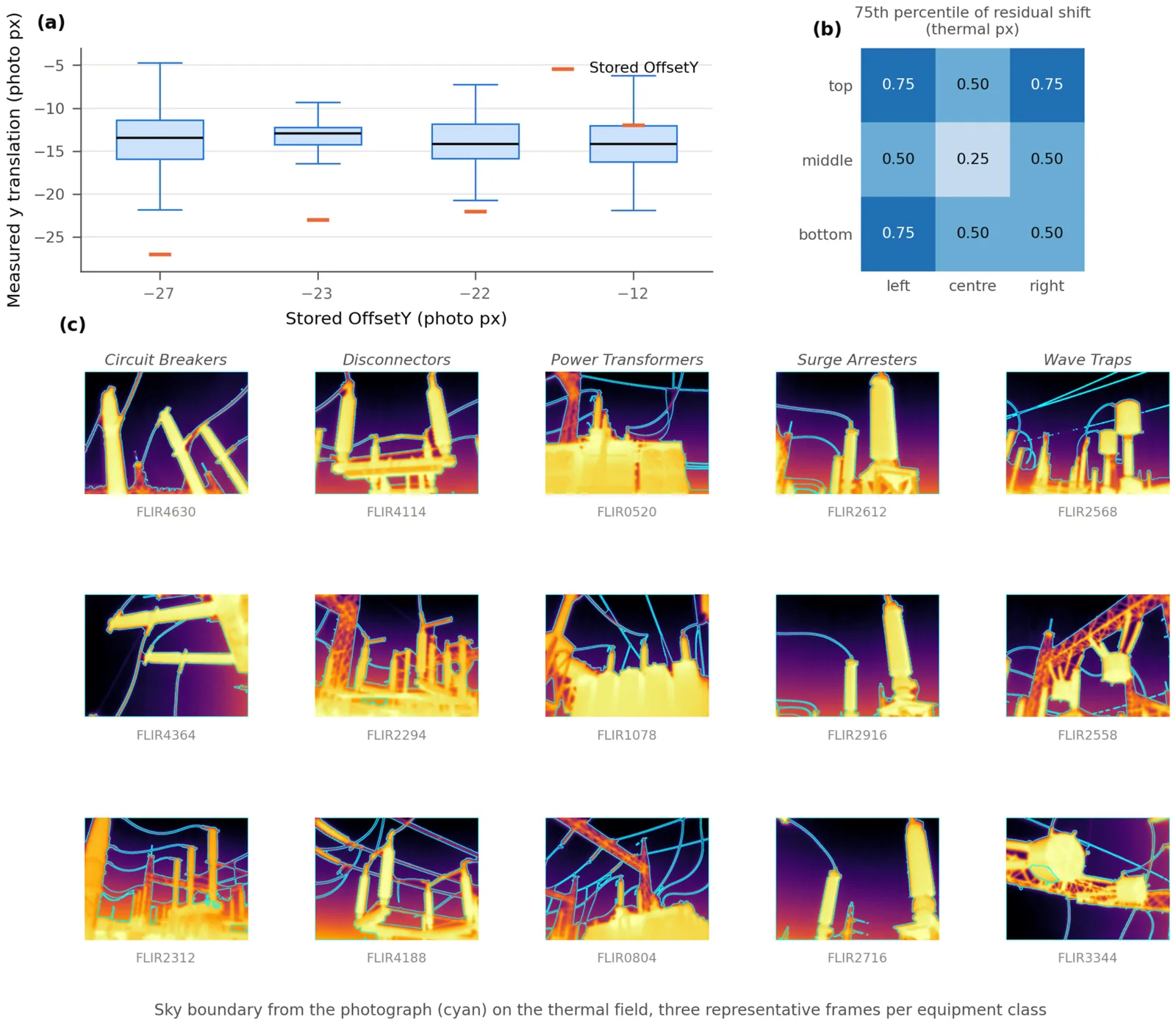
Visible-to-thermal registration. (**a**) Measured translation against the stored OffsetY value. (**b**) Residual shift after per-frame registration, 3 × 3 block grid. (**c**) Recovered sky boundary overlaid on the thermal field, three representative frames per equipment class.

The per-frame translation estimated between the embedded photograph and the thermal grid by maximising the gradient correlation of Equation (5) does not follow the camera’s own stored OffsetY value: the measured translation is statistically indistinguishable across all four distinct stored values, while the stored OffsetX does agree with the measurement (Figure 9a). After per-frame translation, the residual registration error, measured in a 3 × 3 grid of blocks over 300 frames, has a median within ±0.25 thermal pixels in every block (Figure 9b), so no further distortion model was applied. Figure 9c shows the recovered sky-boundary contour overlaid on the thermal field for three representative frames of each of the five equipment classes, confirming that registration quality is consistent within a class and not confined to a single representative example.

**Figure 10:**
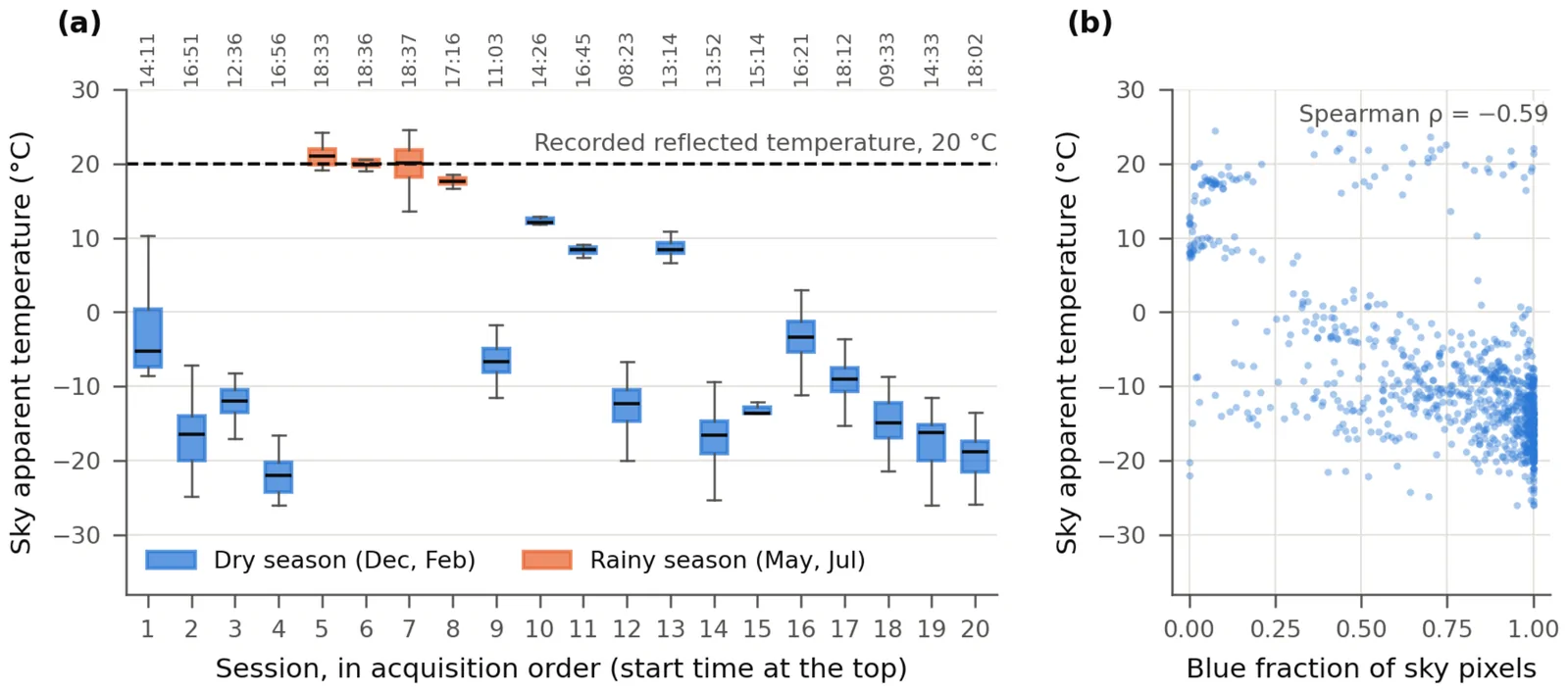
Recovered sky (reflected) temperature. (**a**) By acquisition session and season, against the recorded 20 °C assumption. (**b**) Against the blue fraction of the sky mask.

Figure 10a shows the recovered sky temperature of Equation (6) by acquisition session, grouped by season; the recorded fixed reflected temperature of 20.0 °C lies at or above the upper range of every dry-season session and is exceeded only in three of the four rainy-season sessions. Across the full corpus, the sky temperature is below 20 °C in 18 of 20 sessions and below 0 °C in 13 of them, an error in the recorded assumption of 20 K or more before any material-dependent term is considered. Figure 10b shows that the measured sky temperature correlates with the blue fraction of the sky mask (Spearman ρ = −0.59), consistent with a mix of clear-sky and overcast or hazy conditions across the corpus rather than a single characteristic sky state.

### 3.3. Material Distribution Across the Corpus

**Figure 11:**
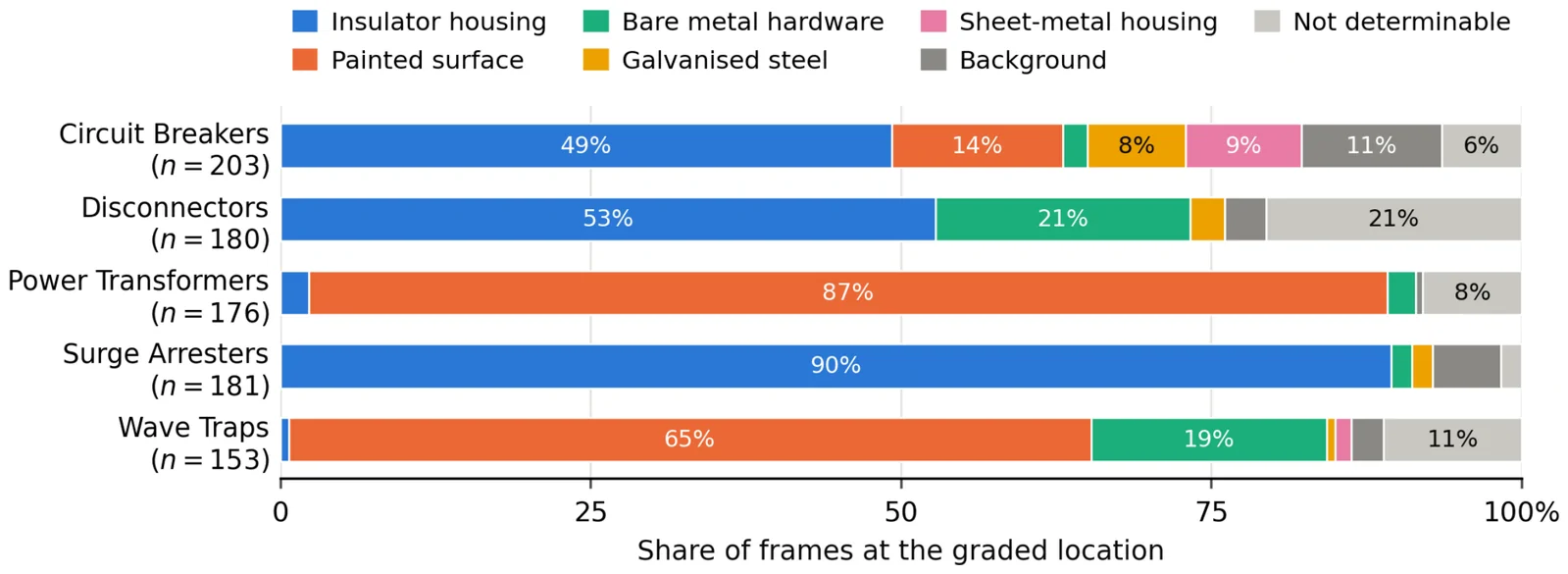
Material distribution at the graded location, by equipment class.

Figure 11 shows the distribution of the seven material codes at the graded location, by equipment class. Insulator housing (40.5%) and painted surfaces (31.4%) together account for 72% of the 893 graded frames; these two materials also carry the tightest emissivity priors (0.90 to 0.95 and 0.85 to 0.95, respectively). Bare metal, galvanised steel and sheet-metal housing together are under 14% of frames by count but, as Section 3.8 shows, dominate the severity uncertainty because their emissivity prior is both lower and wider.

### 3.4. Point-Spread Function and Boundary Contamination

**Figure 12:**
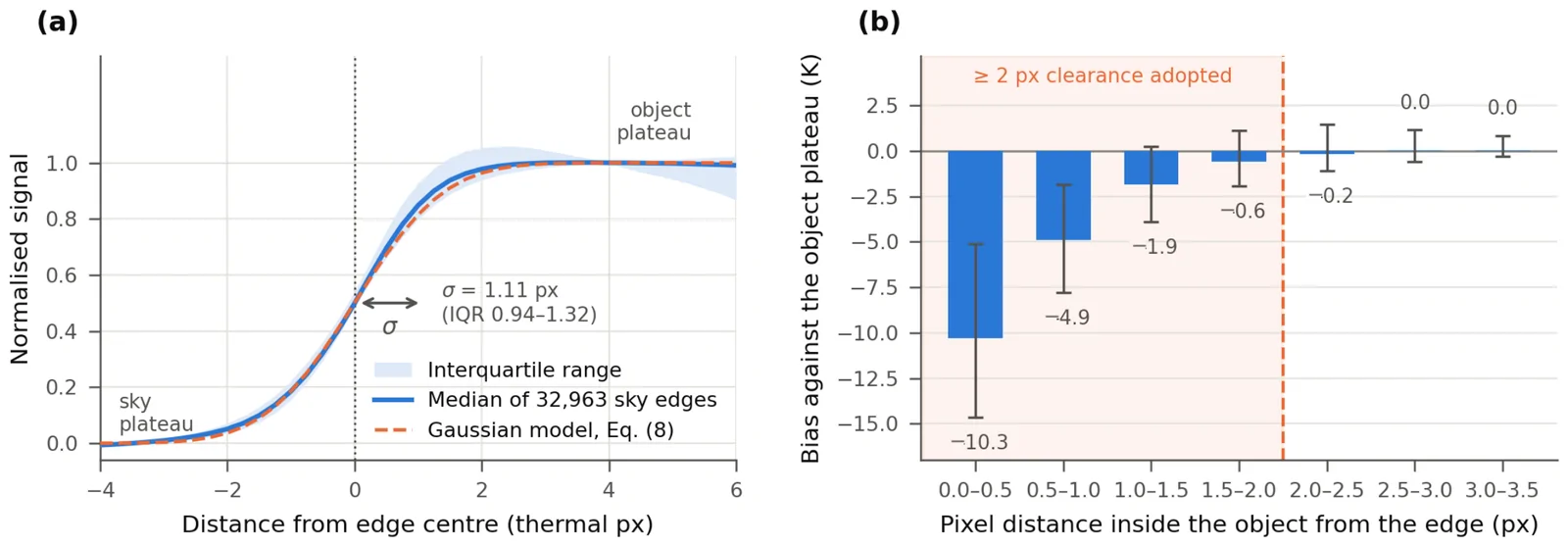
Point-spread and edge contamination. (**a**) Fitted Gaussian edge profile against the measured median profile. (**b**) Measured bias against distance from the edge.

Fitting Equation (8) to 32,963 sampled sky-to-object edges gives a median point-spread sigma of 1.11 thermal pixels (interquartile range 0.94 to 1.32) and a median 10–90% edge width of 2.86 pixels. Figure 12a compares the fitted Gaussian point-spread model against the median measured edge profile, which agree closely across the sampled range. Figure 12b compares the model’s predicted contamination, from Equation (9), against the bias directly measured on 300 sampled frames: 18.5% predicted contamination at one pixel inside the object falls to 3.6% at two pixels and under 0.5% at three pixels, consistent with the measured bias once the local sky-to-object contrast is accounted for. On this basis, both the junction cores of Section 2.6 and the reference surfaces of Section 2.7 were required to stay at least two pixels clear of any sky boundary.

### 3.5. Boundary Continuity Test and Negative Control

**Figure 13:**
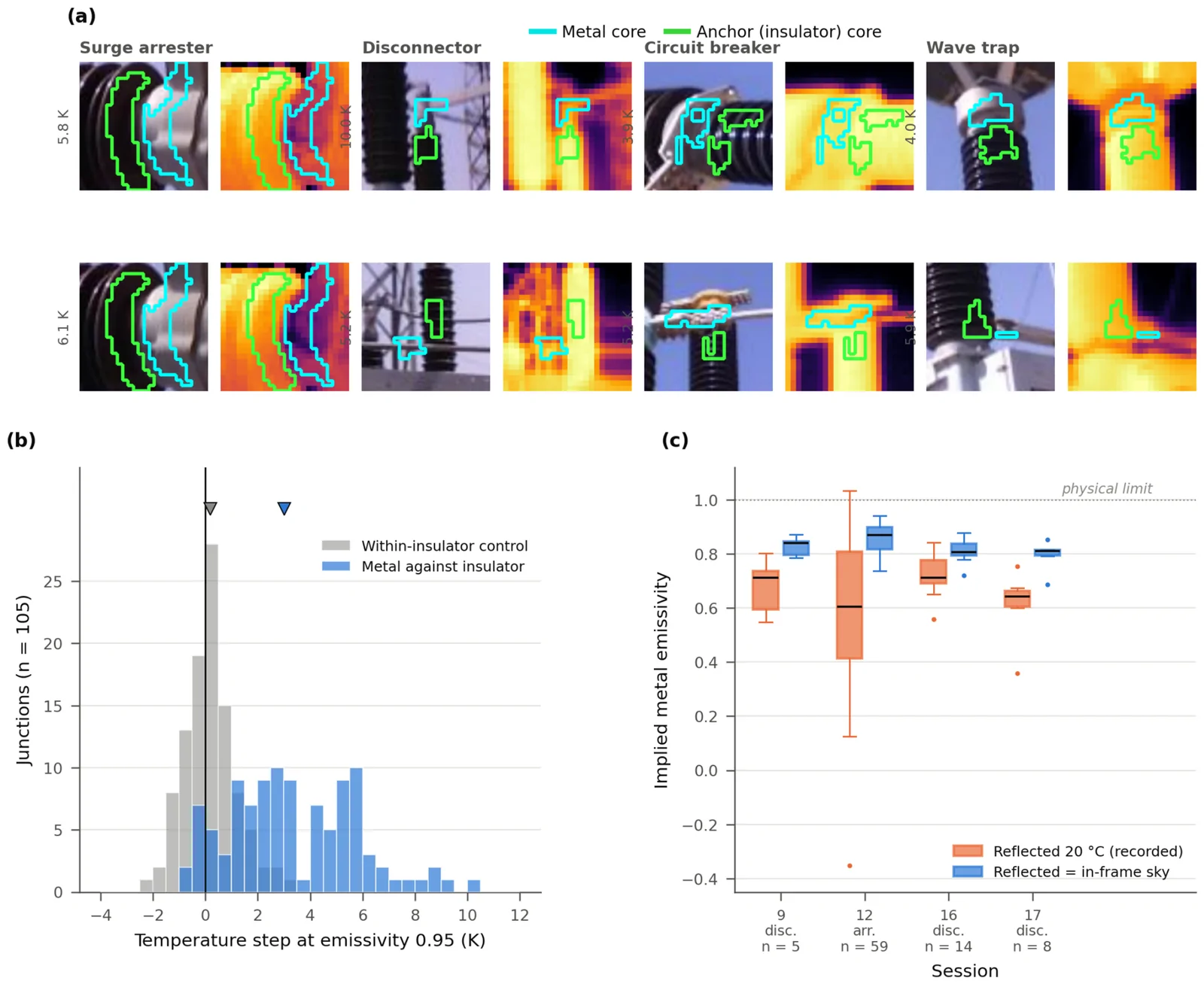
Boundary continuity test. (**a**) Two representative accepted junctions for each equipment class; for wave traps these are the only two accepted junctions in the entire corpus, while power transformers had no accepted junctions and are not shown. (**b**) Temperature-step distribution, metal against insulator versus the within-insulator control. (**c**) Implied metal emissivity by session, recorded versus sky-based reflected temperature.

Of 289 candidate junctions across 173 frames, 106 were accepted by eye review (91 bare-metal fittings, 15 terminals), concentrated in disconnectors, surge arresters and circuit breakers, with few candidates on wave traps and effectively none on power transformers: the geometric segmentation proposed only one candidate boundary on a power transformer frame in the entire corpus, and it was rejected on review, so no power-transformer example can be shown. Figure 13a shows two representative accepted junctions, with their photographic and thermal crops, for each equipment class; wave traps contributed only two accepted junctions in the entire corpus, both on the same frame, and both are shown, while the two shown for surge arresters, disconnectors and circuit breakers are drawn from a larger pool of candidates and are not the only ones available. At the stored default emissivity of 0.95, the metal core reads a median 3.00 K colder than its insulator anchor in 91% of accepted junctions; the within-insulator negative control, sampled 8 to 12 pixels from the same metal core on the same housing, reads a median of only 0.20 K, positive in 58% of cases, consistent with noise around zero (Figure 13b). A Wilcoxon signed-rank test rejects the null hypothesis that the real step and the pseudo-step are drawn from the same distribution at *p* < $10^{-17}$ (n = 105), and separately for the 90 bare-metal fittings (*p* < $10^{-15}$) and the 15 terminals (*p* = 0.0002). The metal-versus-insulator temperature step at a graded junction is therefore a material effect rather than a generic artefact of proximity to a boundary.

### 3.6. Joint Reflected-Radiance Fit

**Figure 14:**
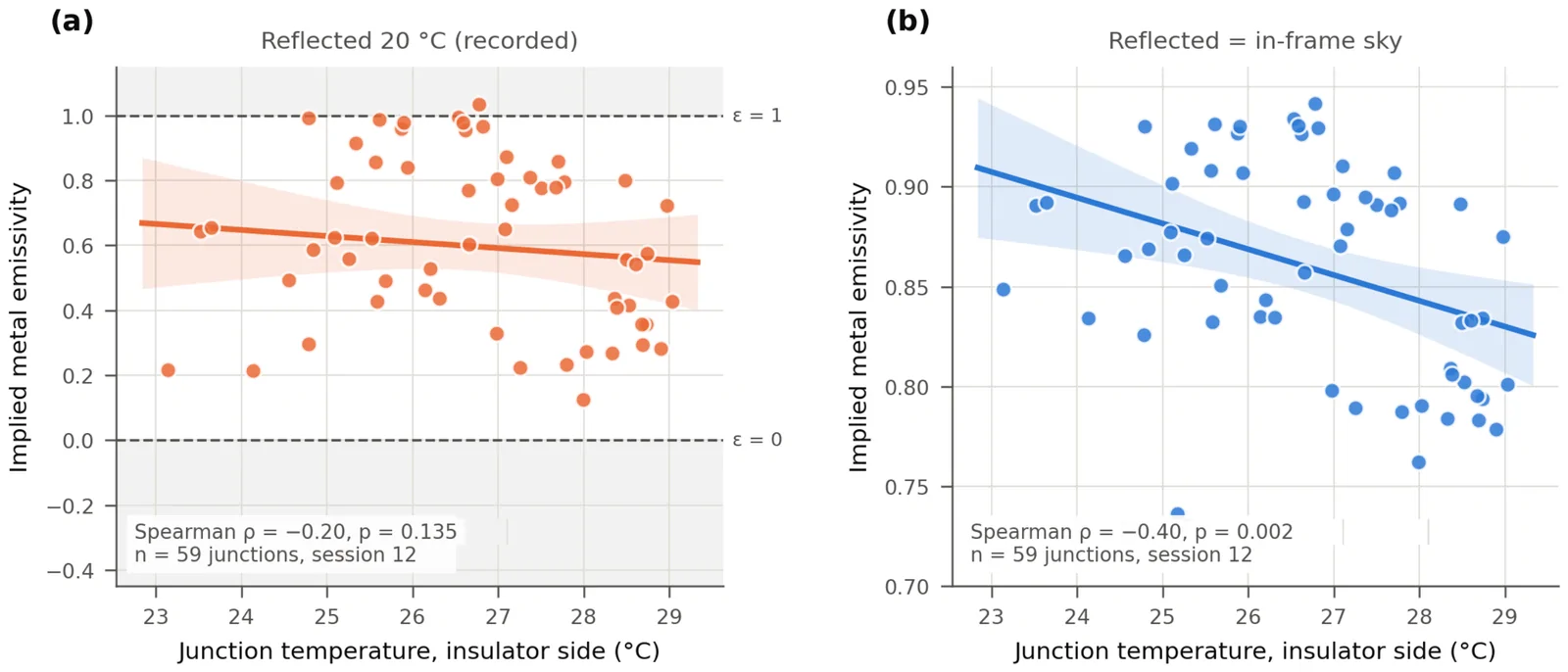
Implied metal emissivity against junction temperature, session 12. (**a**) Under the recorded 20 °C reflected-temperature assumption. (**b**) Under the sky-based (in-frame) reflected-temperature assumption.

Under the recorded 20.0 °C reflected temperature, the closed-form emissivity of Equation (10) gives values as low as 0.12 and occasionally above 1 across the 106 accepted junctions, both physically inadmissible; under the frame’s own measured sky temperature, the same junctions give a credible 0.78 to 0.94 (Figure 13c). The joint fit of Equation (11) resolves cleanly for one session, session 12 (surge arresters, 59 junctions spanning 23.1 to 29.0 °C on the insulator side): emissivity ratio k = 0.370 (bootstrap 95% interval 0.062 to 0.696), metal emissivity 0.34 (0.06 to 0.64), and reflected temperature 22.7 °C (18.6 to 24.6 °C). Figure 14 shows the implied metal emissivity against junction temperature for this session under both reflected-temperature assumptions; the independent drift-scan check (Section 2.6) locates the correlation-crossing reflected temperature at 22 to 24 °C, in close agreement with the joint fit obtained from a different statistical procedure applied to the same data. The other three sessions with enough accepted junctions for a fit (5 to 14 junctions each) span only 1 to 4.5 K in junction temperature, too narrow to constrain the slope individually; pooling those three sessions, which share an equipment class (disconnectors, 27 junctions total), in a fixed-effects fit with a slope shared across sessions narrows the bootstrap interval approximately ninefold relative to the widest individual session’s interval, but the result (metal emissivity 0.60, interval −0.37 to 1.90) still spans outside the physical range [0, 1]. This indicates that the limitation is the volume of same-class junction data available within this corpus rather than a defect of the fitting method itself.

### 3.7. Repeatability Across Independent Views

Of the 106 accepted junctions, 76 form “components” under the consecutive-frame, common-position criterion of Section 2.6, of which 21 components (51 junctions) have two or more independent views. The one-way intraclass correlation of the sky-based emissivity estimate across these repeated views is ICC(1) = 0.51 (within-component standard deviation 0.035, between-component 0.036): approximately half of the variance in nominally repeated measurements of the same physical hardware falls within, rather than between, components. The corresponding figure under the recorded 20.0 °C assumption is numerically higher (ICC 0.57) but is driven by an inflated between-component variance (0.174 against 0.036) that reflects the same reflected-temperature error already shown to bias the point estimate in Section 3.6, and includes individual components whose implied emissivity under this assumption exceeds 1, which is not physical. The sky-based repeatability figure of 0.51 is therefore the more credible estimate of measurement precision, and is carried forward as a stated limitation on the corpus-scale emissivity numbers rather than treated as a defect to be resolved.

### 3.8. Severity Band Assignment Under the Two Criteria

**Figure 15:**
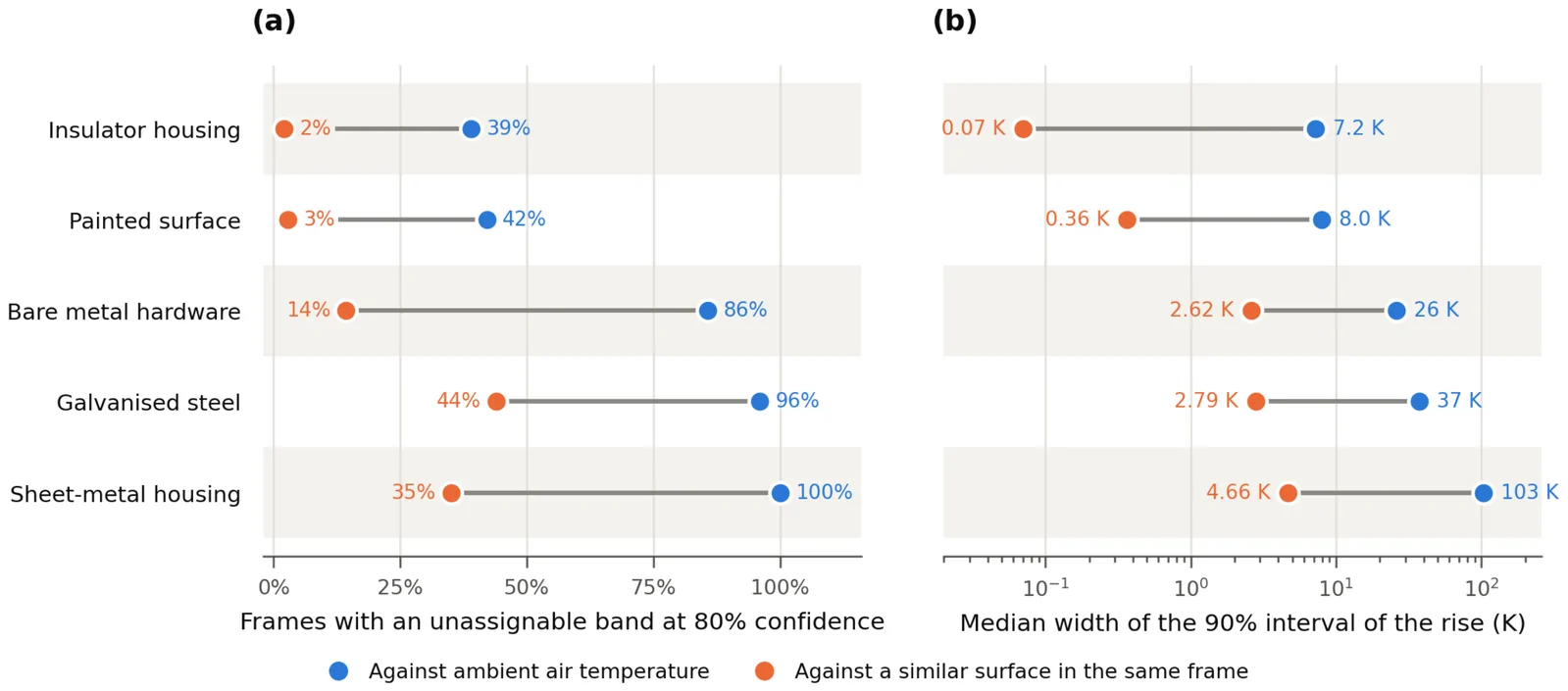
Severity band assignment. (**a**) Fraction of frames with an unassignable band at 80% confidence, by material and criterion. (**b**) Median width of the 90% interval of the rise.

Figure 15 and Table 2 summarise the principal result of this study. Graded against absolute ambient air temperature, even with that air temperature obtained from meteorological reanalysis independently for every session (Section 2.7), the modal severity band cannot be assigned with 80% confidence in 48.4% of the 765 graded frames overall, ranging from 39.0% for insulator housing and 42.1% for painted surfaces up to 85.7% for bare metal, 96.0% for galvanised steel and 100% for sheet-metal housing. Graded instead against a photometrically similar reference surface located elsewhere in the same frame, only 5.9% of frames remain ambiguous overall (2.1% for insulator, 2.8% for paint, 14.3% for bare metal, 44.0% for galvanised, 35.0% for sheet metal), because the reflected-radiance term and, for materials of similar type, much of the emissivity term cancel between the target and its reference. This 5.9% figure holds the reference surface’s own median signal fixed at the value measured for it, matching the Monte Carlo settings used throughout this paper (Section 2.7); Section 3.10 shows that once this reference signal’s own sampling noise is itself propagated as an eighth Monte Carlo input, the relative-criterion fraction rises to 12.2%, with that one source then accounting for the majority of the criterion’s output variance, so 5.9% should be read as a lower bound on the ambiguity remaining under the relative criterion rather than its full uncertainty. Band assignment under the ambient criterion is also unstable to the air-temperature assumption itself: only 2.4% of frames receive the same central-value band across a plausible 20 to 35 °C range, independent of the Monte Carlo spread, directly undermining a single fixed recorded value as a grading basis.

### 3.9. Sensitivity Analysis and Value of Information

**Figure 16:**
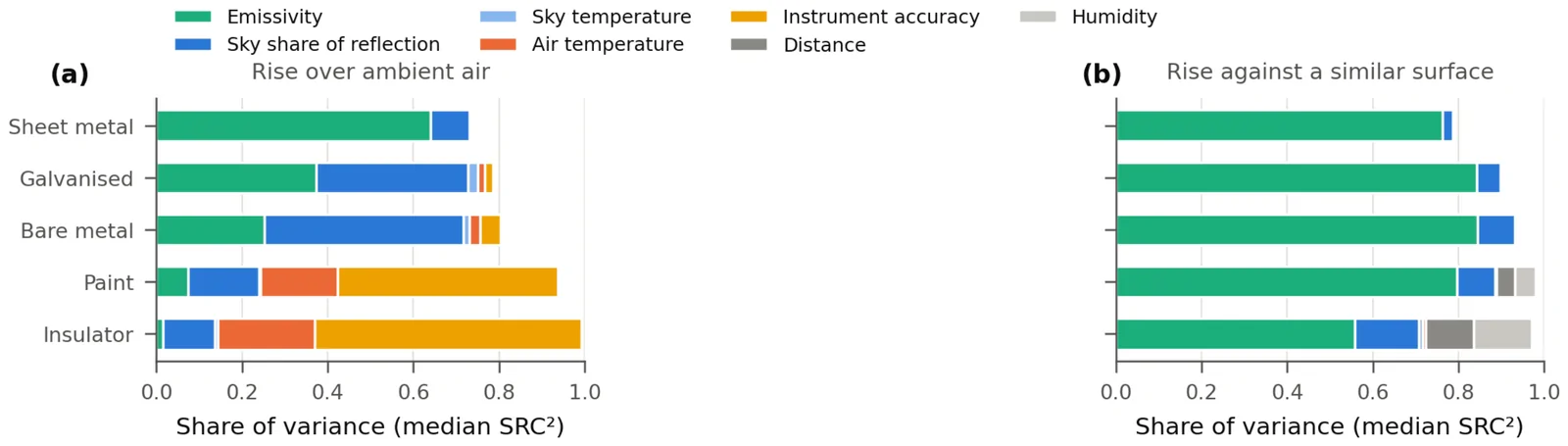
Sensitivity of the temperature rise to each Monte Carlo input (median SRC^2^), by material and criterion. (**a**) Rise over ambient air (the ambient criterion). (**b**) Rise against a similar surface in the frame (the relative criterion).

Figure 16 decomposes the Monte Carlo variance of the temperature rise by input, separately for each material and criterion. For the ambient criterion, the air temperature is obtained from meteorological reanalysis rather than climatological, instrument accuracy is the dominant term for insulators (62% of variance) and painted surfaces (51%), with air temperature a distant second (23% and 18%); for the three metal materials, sky-reflection share and emissivity together account for 72 to 73%. For the relative criterion, emissivity dominates for every material (56 to 85%), since air temperature and, largely, reflected radiance cancel between the target and its reference.

**Figure 17:**
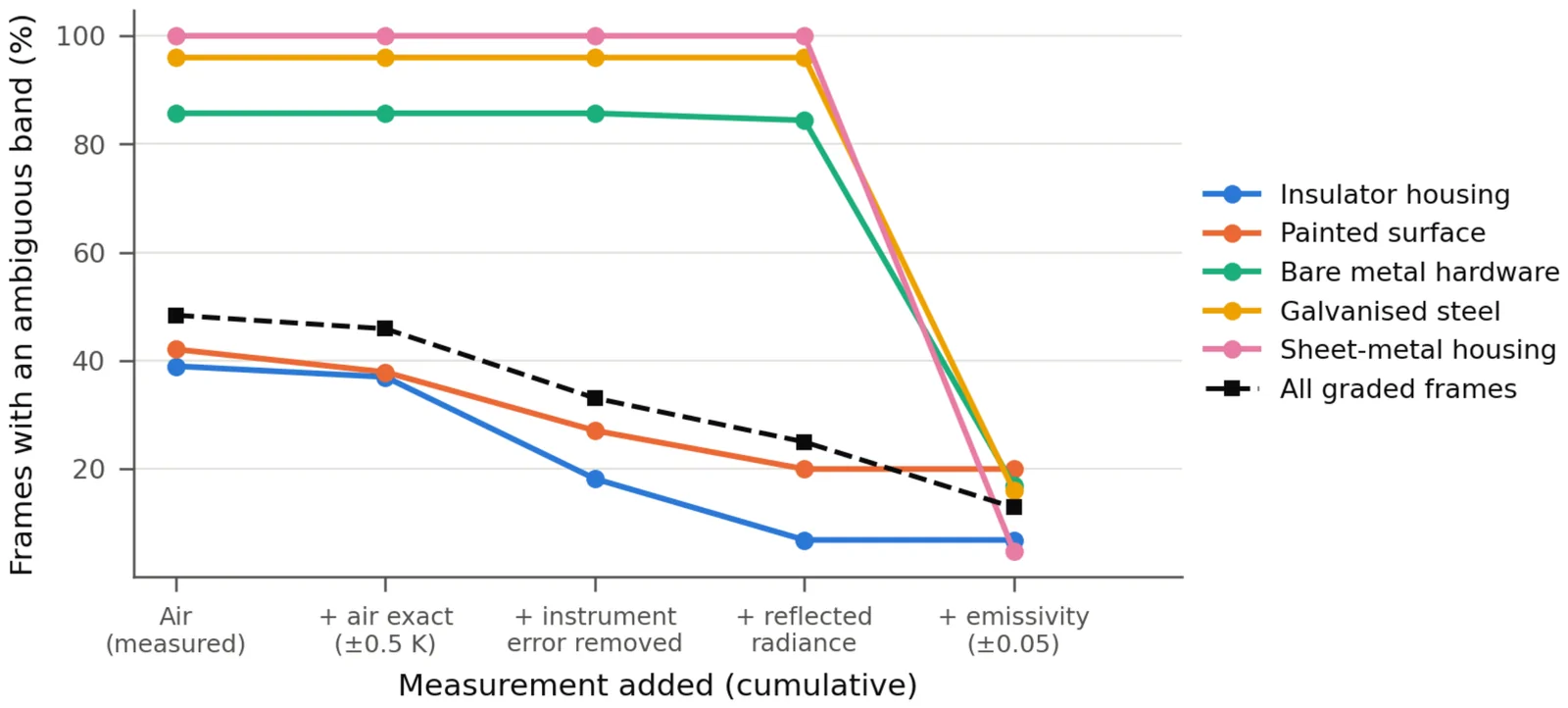
Value of information: ambiguous-band fraction against cumulative measurement, by material.

Figure 17 shows the value-of-information result: starting from the baseline with air temperature obtained from meteorological reanalysis, the ambient-criterion ambiguous fraction falls from 48.4% to 45.9% if air temperature is additionally known to ±0.5 K, to 33.1% once instrument error is excluded, to 25.0% once reflected radiance is also measured, and to 12.9% once emissivity is known to ±0.05 at worst. The pattern differs sharply by material: insulator and paint fall steeply once instrument error and reflected radiance are addressed (to 6.9% and 20.0% respectively) and improve little further from a tighter emissivity prior, while bare metal, galvanised steel and sheet metal remain 84% or more ambiguous until emissivity is constrained, after which they fall to 4.8 to 16.9%. For an inspector without a full radiometric setup, this identifies two distinct priorities rather than one: instrument accuracy and reflected-radiance awareness for insulator- and coating-dominated equipment, and a material-specific emissivity estimate for bare or galvanised metal.

### 3.10. Further Sensitivity and Robustness Analyses

This section extends the sensitivity analysis above along five further dimensions: dependence of the unassignable fractions in Table 2 on the fixed emissivity floor assumed for bare metal and on the 80% confidence threshold itself, availability of a qualifying reference surface for the relative criterion and the contribution of its own sampling noise, the severity-band transitions the correction chain induces at the level of individual frames rather than only in aggregate, and the physical pixel footprint underlying the in-situ emissivity estimate, and the risk that the air temperature used in the ambient criterion is systematically, rather than only randomly, offset from its true value at the graded location. Table 2 and Figure 15, Figure 16 and Figure 17 above are left at the Monte Carlo settings used throughout the rest of this paper, so that the variance decomposition of Figure 16 and the value-of-information cascade of Figure 17 remain internally consistent with the table and figure they were built from; where an analysis below indicates a setting should change, the resulting numbers are reported here as an independently rerun, directly comparable alternative rather than substituted into Table 2 or Figure 15, Figure 16 and Figure 17.

**Reference-surface availability.** The relative criterion requires a photometrically similar reference surface (Section 2.6): a candidate pixel must lie within 30 thermal pixels of the graded location but at least 6 pixels away, be classified as non-sky, carry below 1% boundary contamination, read below 100 °C, and lie within a CIELAB distance of 12 from the target’s own colour in the registered visible image; the reference signal is the median raw signal over at least 20 such pixels, or no reference is returned for that frame. Table 3 gives the resulting availability by material. A qualifying reference was found for 681 of the 765 graded frames overall (89.0%); the relative-criterion figures of Table 2 are computed over this reduced denominator, not over all 765 frames, which is why the Table 2 bare-metal relative-criterion rate is reported against 56 frames rather than the full 77. Bare metal has the highest shortfall (21 of 77 frames, 27.3%), consistent with an isolated metal fitting often lacking a nearby surface similar enough in both distance and colour to qualify; galvanised steel found a qualifying reference in every frame. Among frames with a reference, the number of qualifying candidate pixels is itself variable (median 205, range 22 to 1234).

The relative-criterion figures above also treat this reference surface’s median signal as an exact constant. Re-running the uncertainty propagation of Section 2.7 with the reference median itself bootstrap-resampled from its own qualifying pixels on every Monte Carlo draw, an eighth uncertainty source alongside emissivity, reflected-temperature weighting, sky and air temperature, distance, humidity and instrument noise, raises the relative-criterion overall unassignable fraction from 5.9% to 12.2%, roughly double, and attributes a median 85.5% of the relative criterion’s total output variance across graded frames to reference-surface sampling noise alone, exceeding every other source combined. The reference surface’s own sampling noise is, accordingly, not a second-order correction to the relative criterion but, once it is accounted for, its largest single contributor. We accordingly report 12.2%, not 5.9%, as the relative criterion’s headline figure in the Abstract and Section 3.8; the 5.9% setting is retained only in Table 2 and Figure 15, Figure 16 and Figure 17, so that the variance decomposition of Figure 16 and the value-of-information cascade of Figure 17, both built from that setting, remain internally consistent with the table and figures they describe.

**Sensitivity to a systematic air-temperature offset.** The air-temperature term in the Monte Carlo of Section 2.7 brackets each session’s own reanalysis-based estimate by half the disagreement between two independent reanalysis sources evaluated at the same proxy location (0.75 to 1.55 K either side), which bounds how much the two sources disagree about the weather at that point but not whether the point itself is correct: both sources are queried at a location inferred from the depositors’ institutional affiliation rather than a confirmed substation coordinate (Section 4.3), so a location or local microclimate error shared by both sources would not appear in this bracket at all. The air-temperature-scenario comparison already reported in Section 3.8, in which the central-value ambient band is recomputed at four fixed air temperatures spanning the corpus’s measured range (20, 25, 30 and 35 °C), gives a direct bound on the consequence of exactly this kind of unmodelled offset: of the 765 graded frames, the modal ambient band changes between adjacent 5 °C steps in this range for 42.7% (20 to 25 °C), 59.1% (25 to 30 °C) and 42.4% (30 to 35 °C) of frames, far above the 2.4% of frames stable across the full 15 °C span. A single 5 °C step of this kind already exceeds the 1.5 to 3.1 K bracket the Monte Carlo places around each session’s reanalysis estimate, so an offset of this size is not a risk the existing random term already covers. A systematic offset of this size is well within what an incorrectly located proxy or an unmodelled local thermal environment could plausibly introduce. This risk cannot be bounded further with the data available, since no independent air-temperature measurement at the true substation location exists against which to check the proxy (Section 4.3); it is accordingly carried as an addition to, rather than a component already inside, the ambient criterion’s uncertainty budget, and is a further reason, alongside the terms already in Section 2.7, to prefer the relative criterion over the ambient one.

**Sensitivity to the bare-metal emissivity floor.** Section 2.7’s Monte Carlo prior for bare metal spans 0.45 to 0.90, a floor that sits above the joint-fit point estimate of Section 3.6 (0.34 [0.06, 0.64]). Table 4 sweeps this floor from 0.45, the setting used throughout this paper, down to 0.25, each row an independent rerun of the full severity pipeline; that row reproduces the Table 2 figures to within a few tenths of a percentage point (0 of 893 frames assigned a different ambient modal band, 8 of 893 a different relative modal band, against the Table 2 per-frame file), the residual attributable to the Monte Carlo seed and frame-boundary noise already affecting other reported figures in this paper. Lowering the floor to 0.30, which matches the floor already used for galvanised steel elsewhere in this paper and covers the joint-fit point estimate and its bootstrap interval, raises the ambient-criterion unassignable fraction from 48.4% to 49.0% overall and from 85.7% to 92.2% for bare metal specifically; the relative criterion is materially unaffected (5.9% to 6.3% overall; 14.3% to 14.3% for bare metal, unchanged within rounding), because the reflected-radiance and emissivity terms the floor perturbs are largely shared between a bare-metal target and its reference and therefore cancel. The qualitative conclusion of Section 3.8, that the ambient criterion leaves most metal targets unassignable while the relative criterion does not, holds at every floor in Table 4.

**Sensitivity to the confidence threshold.** Table 2 fixes the confidence threshold for “unassignable” at 80%. Table 5 sweeps this threshold from 50% to 95% at the 0.30 floor of Table 4, showing the ambient criterion’s unassignable fraction climbing continuously across the whole range rather than turning sharply at any one value, so the 80% threshold used throughout this paper is a conventional choice on a smooth curve, not a value that happens to sit at a discontinuity.

**Severity-band transitions under the chain correction.** Table 2 and Figure 15 report the ambient and relative criteria only after the full correction chain; Table 6 instead cross-tabulates each frame’s severity band under the camera’s as-recorded settings against its band under the corrected chain, to show the decision changing directly rather than only in aggregate. Switching from the camera’s as-recorded settings to the corrected chain changes the assigned band for 79.6% of the 765 graded frames under the ambient criterion and for 4.3% of the 681 frames with a qualifying reference under the relative criterion.

**Pixel footprint and junction-core separation.** The FLIR C5’s field of view (54° × 42° over 160 × 120 px) gives, at the corpus’s recorded 10 m object distance, a single-pixel footprint of 58.9 mm horizontally by 61.1 mm vertically. The accepted junctions’ metal and anchor cores, two to six pixels on either side of the boundary (Section 2.6), accordingly sit within a physical separation of roughly 118 to 353 mm, the spatial scale over which the isothermal-junction assumption of Equation (10) must hold. The gradient-sensitivity analysis above quantifies the consequence of that assumption: across all 106 accepted junctions, the gap between the junction temperature and the assumed reflected temperature is never smaller than 35.5 K, so even an unmodelled gradient of several kelvin between the two cores biases the recovered emissivity by at most a few hundredths, and the existing accepted set is not further restricted on this basis. The Monte Carlo’s U(3, 15) m distance prior (Section 2.7) scales this physical footprint by the same factor at its extremes, a factor of 5 between 15 m and 3 m, independently of that prior’s effect on the transmittance term τ.

## 4. Discussion

### 4.1. Principal Findings

The central finding of this paper is an asymmetry, not a uniform correction. Once emissivity, reflected radiance and air temperature are all estimated rather than assumed outright, the criterion conventionally used in field practice, a rise graded against ambient air temperature, remains unable to support a severity call for the majority of non-insulator, non-paint targets and for approximately two in five insulator and paint frames. A criterion graded instead against a similar surface elsewhere in the same frame is robust across materials throughout, because it depends far less on the two terms that are most difficult to determine in the field, reflected radiance and emissivity, which largely cancel between a target and a photometrically similar reference. This suggests that the practical value of the correction developed in this paper is not primarily a revised absolute-temperature number, but evidence for which grading criterion a given piece of equipment should be read against, and which single additional measurement is worth the operator’s time for a given material.

### 4.2. Comparison with Prior Emissivity-Correction Studies

Both the nearest prior works, Han et al. [70] and Klingspon et al. [12], establish visible-channel segmentation as a workable route to per-material emissivity correction, and this paper adopts the same general strategy. The present contribution differs in three respects. First, validation here rests on physical constraints internal to the corpus itself, a within-insulator negative control that can and would fail if the observed step were an artefact, and a joint fit that resolves independently of any assumed reflected temperature, rather than on a single laboratory thermocouple reading [70] or a synthetic benchmark and a high-emissivity control [12]. The width of the uncertainty budget obtained in Section 2.7 is also broadly consistent with the general error models of Ferreira et al. [50] and Playà-Montmany and Tattersall [51], drawn, respectively, from non-substation electrical and ecological infrared applications, which supports treating the emissivity- and reflected-radiance-driven spread as a property of conventional field thermography in general rather than an artefact specific to this corpus. Second, this paper reports where its own method does not resolve, stating a repeatability limit of ICC ≈ 0.51 and a joint fit that succeeds for one session of four attempts, rather than presenting corpus-scale emissivity numbers as exact. Third, this paper carries the correction through to its consequence for a maintenance decision under an explicit uncertainty budget, which neither prior work attempts.

### 4.3. Limitations

Several limitations bound the scope of these results. Accepted junctions concentrate in disconnectors, surge arresters and circuit breakers, with almost none on power transformers, so the boundary validation of Section 3.5 and the joint fit of Section 3.6 apply directly to a subset of equipment and sessions. This class imbalance was checked rather than assumed: applying the same geometric segmentation without the graded-location restriction, over every quality-passing power transformer frame, raised the candidate count from one to approximately 2700, but visual review of the largest candidates showed shadowed radiator fins, corrugated tank cladding and ladder structure rather than genuine metal-to-insulator adjacency. Power transformer frames in this corpus are dominated by large painted-steel tank and radiator surfaces, in which shadow rather than material change is the principal source of the local darkness the segmentation uses to flag a housing region, so the absence is attributable to scene composition rather than to a segmentation threshold or a shortage of candidates. Elsewhere the severity propagation of Section 2.7 relies on the measured sky temperature used directly rather than jointly fitted with emissivity. The anchor emissivity for glazed porcelain (0.92) is taken from established reference values rather than measured for this corpus in the relevant 8 to 14 μm band. The repeatability of the corpus-scale emissivity estimate is moderate rather than tight (ICC(1) ≈ 0.51), which should be read as a stated bound on the precision of any single component’s emissivity rather than a defect to be resolved by more data alone, though the pooled fit of Section 3.6 indicates that more same-class junctions specifically would narrow it further. The air temperature used in the ambient criterion, obtained independently for every session from meteorological reanalysis rather than drawn from a climatology, is itself drawn from a reanalysis grid cell at a proxy location inferred from the depositors’ institutional affiliation rather than a confirmed substation coordinate, and the two independent reanalysis sources used disagree by a median of 2.6 K and a maximum of 6.2 K, which is the only available check on that proxy. Section 3.10 links this risk directly to the severity-band conclusions: a systematic offset no larger than a single 5 °C step between the fixed air-temperature scenarios already used in Section 3.8 changes the modal ambient band for 42 to 59% of graded frames, a step itself larger than the 1.5 to 3.1 K bracket the Monte Carlo already carries for reanalysis-source disagreement in Section 2.7. Finally, the local segmentation used to find junction boundaries is a targeted, geometric procedure restricted to the graded location and its immediate boundary, not a general-purpose material segmenter evaluated by intersection-over-union; this was sufficient to reach the results reported here and is not put forward as a substitute for one.

Two further physical dependencies of emissivity are not modelled. First, emissivity assigned here depends only on material, not on viewing angle, although many components in this corpus (bushings, arrester housings, conductors) are cylindrical and are imaged from varying angles of incidence as the operator moves around the equipment; direct measurements of the calibration error introduced by high angles of view in infrared thermography [75], a correction specifically for curved-surface targets under the irradiation-reflection method [76], and a visible-image-assisted method for characterising this directional dependence of longwave radiation on building equipment [77], all report errors of a comparable order to the uncertainty budget already carried through Section 2.7, but this corpus does not record the viewing angle at each graded location, so the effect cannot be quantified or corrected retrospectively here. Second, the material-dependent emissivity priors of Section 2.7 assume a dry surface; outdoor equipment cooled below the dew point by radiative loss to a cold sky can carry a condensation layer whose effective emissivity, in the earliest moments of droplet formation particularly, can shift substantially towards that of water rather than the underlying material [78]. The relative-humidity term already in the Monte Carlo (Section 2.7) is at best a crude proxy for this risk, since it does not model the dew-point crossing itself, and per-frame surface-wetness information is not available for this corpus, so which specific frames, if any, were affected cannot be determined retrospectively.

This evaluation is also bounded by what a single corpus and a single camera model can show: all 893 frames come from one consumer-grade radiometric camera (FLIR C5) and one set of acquisition parameters applied uniformly across every session (Section 2.1), and the method is not itself tested here against other camera hardware, outdoor environments or plant configurations. The radiometric chain is not hardcoded to this camera, however: the Planck-type calibration constants and atmospheric-transmission coefficients used throughout (Section 2.2) are read from each frame’s own embedded metadata rather than fixed for the FLIR C5, so the same correction would run unchanged on any FLIR camera that records per-frame calibration data alongside a co-registered visible channel in this way. What would not transfer without recalibration are the corpus-specific choices made here: the material-dependent emissivity priors of Section 2.7, fitted to the five equipment classes and surface finishes present in this corpus, and the acceptance thresholds for a candidate junction and a qualifying reference surface (Section 2.6), set against this corpus’s own pixel scale and image quality. Applying the method to a different camera, site, or equipment mix would need its own priors and thresholds recalibrated before the severity-band conclusions of Section 3.8 could be expected to transfer, which Section 5 identifies as future work.

## 5. Conclusions

This paper corrected and validated, without a reference thermometer, the single largest simplifying assumption in field infrared inspection of substation equipment: that one emissivity and one reflected temperature describe every material in every frame. Using only the co-registered visible channel already recorded by a modern radiometric camera, in-situ emissivity was estimated at 106 material boundaries across four equipment classes, validated against a within-insulator negative control (*p* < $10^{-17}$) and, where the data allowed, against an independently fitted reflected temperature, and found to be repeatable to within an intraclass correlation of 0.51 across independent views of the same hardware. Propagating this and the other dominant sources of measurement uncertainty through 765 graded frames shows that the conventional ambient-air severity criterion remains unable to support a maintenance call for the majority of metal targets and for a substantial minority of insulator and painted targets, even once air temperature is itself obtained from meteorological reanalysis rather than assumed, while a criterion graded against a similar surface in the same frame is robust throughout. A value-of-information ranking further shows that the most useful single additional measurement is material-dependent: instrument accuracy and reflected-radiance awareness for insulator- and coating-dominated equipment, and a material-specific emissivity estimate for bare or galvanised metal.

More broadly, this correction treats a handheld radiometric camera as a proximal thermal remote-sensing instrument rather than a fixed-setting tool: the same measurement equation, and the same emissivity term most often assumed rather than measured, underlies land surface temperature retrieval at the satellite and airborne scale. Recovering a per-pixel emissivity and reflected radiance from a second, co-registered channel, in place of the operator’s single session-wide default, is accordingly not a device specific to substation inspection, and the value-of-information ranking obtained here offers a template for prioritising measurement effort in other close-range thermal remote-sensing settings built on the same reflected-radiance and emissivity ambiguity.

Future work includes extending the joint reflected-radiance fit as further same-class junction data become available, and evaluating whether the relative grading criterion validated here transfers to substation corpora acquired at other sites and with other camera models, and more generally, to other proximal or close-range thermal remote-sensing applications built on the same measurement equation.

## Figures and Tables

**Table 1:** Difference between three candidate recoveries and the camera’s own burnt-in readouts (802 plain readouts).

Recovery	Median Signed Difference (K)	Median Absolute Difference (K)	Frames Within 0.1 K (%)	Frames Within 0.5 K (%)
Direct inversion, Equation (1) only (no atmospheric term)	−1.17	1.27	0.7	8.7
Split-path, τ(d/2)^2^ [60]	+0.15	0.20	17	81
Whole-path, τ(d) (this study)	−0.01	0.06	58	82

**Table 2:** Frames with an unassignable severity band (80% confidence threshold) and the median width of the 90% interval of the rise, by material and criterion (765 graded frames).

Material	Ambient: Unassignable	Ambient: 90% Interval Width (K)	Relative: Unassignable	Relative: 90% Interval Width (K)
Insulator housing	39.0%	7.2	2.1%	0.1
Painted surface	42.1%	8.0	2.8%	0.4
Bare metal	85.7%	25.9	14.3%	2.6
Galvanised steel	96.0%	37.5	44.0%	2.8
Sheet-metal housing	100%	102.7	35.0%	4.7
All graded frames	48.4%	n/a	5.9%	n/a

**Table 3:** Availability of a qualifying reference surface for the relative criterion, by material (765 graded frames).

Material	Graded Frames	No Qualifying Reference	No-Reference Rate
Insulator housing	362	32	8.8%
Painted surface	280	30	10.7%
Bare metal	77	21	27.3%
Galvanised steel	25	0	0.0%
Sheet-metal housing	21	1	4.8%
All graded frames	765	84	11.0%

**Table 4:** Sensitivity of the severity-band unassignable fraction to the bare-metal emissivity floor (ceiling fixed at 0.90; 0.45 is the setting used throughout this paper, 0.30 the floor matching galvanised steel elsewhere in this paper).

Bare-Metal Floor	Ambient: Overall	Ambient: Bare Metal	Relative: Overall	Relative: Bare Metal
0.45 (used throughout this paper)	48.4%	85.7%	6.0%	10.7%
0.40	48.5%	87.0%	6.2%	12.5%
0.34 (joint-fit point estimate)	48.8%	89.6%	6.3%	14.3%
0.30	49.0%	92.2%	6.3%	14.3%
0.25	49.2%	93.5%	6.8%	19.6%

**Table 5:** Severity-band unassignable fraction against the confidence threshold, at the 0.30 bare-metal emissivity floor.

Confidence Threshold	Ambient: Overall	Ambient: Bare Metal	Relative: Overall	Relative: Bare Metal
50%	13.1%	74.0%	0.0%	0.0%
60%	26.3%	79.2%	1.9%	3.6%
70%	38.6%	84.4%	4.1%	12.5%
80% (used throughout this paper)	49.0%	92.2%	6.3%	14.3%
85%	56.9%	94.8%	7.8%	19.6%
90%	70.7%	96.1%	9.7%	28.6%
95%	83.8%	97.4%	10.7%	35.7%

**Table 6:** Frames whose severity band changes between the camera’s as-recorded settings and the corrected chain, by material, ambient criterion (765 graded frames).

Material	Graded Frames	Band Changes
Insulator housing	362	81.2%
Painted surface	280	75.7%
Bare metal	77	87.0%
Galvanised steel	25	84.0%
Sheet-metal housing	21	71.4%
All graded frames	765	79.6%

## Data Availability

The source imagery analysed in this study is a publicly deposited third-party dataset [68], available from Science Data Bank under a CC BY-NC-SA 4.0 licence. The code, intermediate results and material-labelling and junction-review sheets generated in this study are available from the corresponding author on reasonable request.

## References

[1] InterNational Electrical Testing Association (2023). ANSI/NETA MTS-2023: Standard for Maintenance Testing Specifications for Electrical Power Equipment and Systems.

[2] Minkina W., Gryś S. (2024). Thermographic Measurements in Electrical Power Engineering—Open Discussion on How to Interpret the Results. Appl. Sci..

[3] Xia C., Ren M., Wang B., Dong M., Xu G., Xie J., Zhang C. (2021). Infrared thermography-based diagnostics on power equipment: State-of-the-art. High Volt..

[4] Minkina W., Dudzik S. (2009). Infrared Thermography: Errors and Uncertainties.

[5] Vollmer M., Möllmann K.-P. (2017). Infrared Thermal Imaging: Fundamentals, Research and Applications.

[6] Teledyne FLIR LLC (2023). FLIR C5 Compact Thermal Camera: Technical Datasheet.

[7] Andrade A.F., Costa E.G., Fernandes J.M.B., Alves H.M.M., Amorim Filho C.R.C. (2019). Thermal behaviour analysis in a porcelain-housed ZnO surge arrester by computer simulations and thermography. High Volt..

[8] Der B., Raszková S., Wald F., Bihina G., Gaigl C., Rus V., Malaska M. (2022). Emissivity of hot-dip galvanized surfaces in future development of EN 1993-1-2. J. Struct. Fire Eng..

[9] Riba J.-R., Liu Y., Moreno-Eguilaz M. (2024). Analyzing the role of emissivity in stranded conductors for overhead power lines. Int. J. Electr. Power Energy Syst..

[10] Riba J.-R., Soni A., Moreno-Eguilaz M. (2025). Emissivity-temperature dependence of Cu and Al conductors up to about 250 °C and its effects. Sens. Actuators A Phys..

[11] Suesut T., Nunak N., Nunak T., Rotrugsa A., Tuppadung Y. Emissivity measurements on material and equipment in electrical distribution system. Proceedings of the 2011 8th International Conference on Electrical Engineering/Electronics, Computer, Telecommunications and Information Technology (ECTI-CON).

[12] Klingspon J., McAvoy S., Seracini M., Kuester F. (2026). Material-Segmented Per-Pixel Emissivity Correction for Thermographic Anomaly Detection in Cultural Heritage Digital Twins. arXiv.

[13] Gillespie A., Rokugawa S., Matsunaga T., Cothern J.S., Hook S., Kahle A.B. (1998). A temperature and emissivity separation algorithm for Advanced Spaceborne Thermal Emission and Reflection Radiometer (ASTER) images. IEEE Trans. Geosci. Remote Sens..

[14] Wang S., He L., Hu W. (2015). A Temperature and Emissivity Separation Algorithm for Landsat-8 Thermal Infrared Sensor Data. Remote Sens..

[15] Ding D.-H., Fang K., Yu H.-X., Qian K.-J., Chen Y.-N., Cui D.-J. (2014). Multi-emissivity setting in thermal imaging based on visible-light image segmentation. TELKOMNIKA Indones. J. Electr. Eng..

[16] Gao Y., Tian G.Y. (2018). Emissivity correction using spectrum correlation of infrared and visible images. Sens. Actuators A Phys..

[17] Schmid S., Seitz L., Menéndez Orellana A.E., Grosse C.U. (2025). Correction of emissivity in thermograms with neural networks. Quant. InfraRed Thermogr. J..

[18] Toullier T., Dumoulin J., Mevel L. (2025). New joint estimation method for emissivity and temperature distribution based on a Kriged Marginalized Particle Filter. Sci. Remote Sens..

[19] Rodríguez S., Keshmiri S., Butt H., Fleck B., Qureshi A. (2026). Modeling for Thermal Data Correction in Emissivity-Variant Materials: Numerical and Machine Learning Approaches. J. Manuf. Mater. Process..

[20] Mlačnik J., Pušnik I. (2026). A Comparison of Radiometric and Spectrometric Emissivity Evaluation Methods in Infrared Thermometry. Sensors.

[21] Ennass K., Remy D., Schick V., Meulemans D. (2025). Multi-spectral infrared thermography using finite element emissivity modelling for high-temperature measurements on metallic surfaces. Infrared Phys. Technol..

[22] Yang Y., Bai L., Zheng W., Wang X. (2025). A data processing method for multispectral emissivity and temperature with the introduction of new objective function and nonlinear constraints. Opt. Commun..

[23] Kim J., Song D. (2025). Quantitative micrometer-scale heat dissipation analysis using pixel-level emissivity correction-based operando IR thermography. Case Stud. Therm. Eng..

[24] Li Z.-L., Tang B.-H., Wu H., Ren H., Yan G., Wan Z., Trigo I.F., Sobrino J.A. (2013). Satellite-derived land surface temperature: Current status and perspectives. Remote Sens. Environ..

[25] Kruczek T. (2023). Conditions for use of long-wave infrared camera to measure the temperature of the sky. Energy.

[26] Toullier T., Dumoulin J. (2024). Bias and bottlenecks study in outdoor long term thermal monitoring by infrared thermography under variable atmospheric and illumination conditions. Infrared Phys. Technol..

[27] Lafargue-Tallet T., Vaucelle R., Caliot C., Aouali A., Abisset-Chavanne E., Sommier A., Peiffer R., Pradere C. (2022). Active thermo-reflectometry for absolute temperature measurement by infrared thermography on specular materials. Sci. Rep..

[28] Adán A., Pérez V., Ramón A., Castilla F.J. (2023). Correction of Temperature from Infrared Cameras for More Precise As-Is 3D Thermal Models of Buildings. Appl. Sci..

[29] Silva Junior O., Coninck J.C.P., Magrin F.G.S., Ganacim F.I.S., Pombeiro A., Fernandes L.G., Romaneli E.F.R. (2023). Impacts of Atmospheric and Load Conditions on the Power Substation Equipment Temperature Model. Energies.

[30] Zhang Y., Wang B., Tang F. (2026). Regional Infrared and Visible Image Registration for Distribution Substation Areas with Wide Depth of Field. High Volt..

[31] Shao Y., Vasilev A.S. (2026). A Cost-Effective and Rapidly Manufacturable Infrared–Visible High-Contrast Calibration Board Based on Structural Parametrization. J. Imaging.

[32] Güidi N.E., di Filippo A., Barba S. (2025). High-Resolution 3D Thermal Mapping: From Dual-Sensor Calibration to Thermally Enriched Point Clouds. Appl. Sci..

[33] Meng L., Zhou J., Liu S., Wang Z., Zhang X., Ding L., Shen L., Wang S. (2022). A robust registration method for UAV thermal infrared and visible images taken by dual-cameras. ISPRS J. Photogramm. Remote Sens..

[34] Chen N., Yang X., Chang H. (2026). Image splicing and semantic segmentation-based omni-directional monitoring for substation equipment. Discov. Artif. Intell..

[35] Liu Y., Wu Z., Song M., Wu W. (2026). Development of a feature differentiation fusion semantic segmentation model (DFF-SSN) for robust substation visual perception. Intell. Build. Int..

[36] Gomes A.A.P., Ganacim F.I.S., Magrin F.G.S., Bobko N., Fernandes L.G., Pombeiro A., Romaneli E.F.R. (2023). A Semantically Annotated 15-Class Ground Truth Dataset for Substation Equipment to Train Semantic Segmentation Models. Data.

[37] Liu J., Xu C., Ye Q., Cao L., Dai X., Li Q. (2024). Thermal Imaging-Based Abnormal Heating Detection for High-Voltage Power Equipment. Energies.

[38] Wang F., Guo Y., Li C., Lu A., Ding Z., Tang J., Luo B. (2022). Electrical Thermal Image Semantic Segmentation: Large-Scale Dataset and Baseline. IEEE Trans. Instrum. Meas..

[39] Li Z., Zhang Y., Wu H., Suzuki S., Namiki A., Wang W. (2023). Design and Application of a UAV Autonomous Inspection System for High-Voltage Power Transmission Lines. Remote Sens..

[40] Chatzargyros G., Papakonstantinou A., Kotoula V., Stimoniaris D., Tsiamitros D. (2024). UAV Inspections of Power Transmission Networks with AI Technology: A Case Study of Lesvos Island in Greece. Energies.

[41] Tanda G., Migliazzi M. (2024). Infrared thermography monitoring of solar photovoltaic systems: A comparison between UAV and aircraft remote sensing platforms. Therm. Sci. Eng. Prog..

[42] Zarco-Periñán P.J., Martínez-Ramos J.L., Zarco-Soto F.J. (2021). A novel method to correct temperature problems revealed by infrared thermography in electrical substations. Infrared Phys. Technol..

[43] Zarco-Periñán P.J., Zarco-Soto F.J., Zarco-Soto I.M., Martínez-Ramos J.L. (2022). Conducting Thermographic Inspections in Electrical Substations: A Survey. Appl. Sci..

[44] (2018). Standard Guide for Examining Electrical and Mechanical Equipment with Infrared Thermography.

[45] (2008). Condition Monitoring and Diagnostics of Machines—Thermography—Part 1: General Procedures.

[46] Dziarski K., Hulewicz A. (2021). Uncertainty of Thermographic Temperature Measurement with an Additional Close-up Lens. Meas. Sci. Rev..

[47] Dziarski K., Hulewicz A. (2021). Uncertainty of Thermographic Temperature Measurement of Electric Units Contained in Switchgear. Pomiary Autom. Robot..

[48] Hulewicz A., Dziarski K., Krawiecki Z. (2023). The Estimated Temperature of the Semiconductor Diode Junction on the Basis of the Remote Thermographic Measurement. Sensors.

[49] García-López C., Álvarez-Tey G. (2022). Evaluation of the Uncertainty of Surface Temperature Measurements in Photovoltaic Modules in Outdoor Operation. Sensors.

[50] Ferreira R.A.M., Silva B.P.A., Teixeira G.G.D., Andrade R.M., Porto M.P. (2019). Uncertainty analysis applied to electrical components diagnosis by infrared thermography. Measurement.

[51] Playà-Montmany N., Tattersall G.J. (2021). Spot size, distance and emissivity errors in field applications of infrared thermography. Methods Ecol. Evol..

[52] (2008). Evaluation of Measurement Data—Supplement 1 to the “Guide to the Expression of Uncertainty in Measurement”—Propagation of Distributions Using a Monte Carlo Method.

[53] Saltelli A., Annoni P., Azzini I., Campolongo F., Ratto M., Tarantola S. (2010). Variance based sensitivity analysis of model output. Design and estimator for the total sensitivity index. Comput. Phys. Commun..

[54] Sedivy S., Remek L., Kozel M., Sramek J., Mikolaj J. (2026). Application of Value of Information-Based Approaches in Road Inspection Processes and Asset Management: A Literature Review. Infrastructures.

[55] Liu X., Wang P. (2022). Valuation of Continuous Monitoring Systems for Engineering System Design in Recurrent Maintenance Decision Scenarios. J. Mech. Des..

[56] Minkina W., Klecha D. (2016). Atmospheric transmission coefficient modelling in the infrared for thermovision measurements. J. Sens. Sens. Syst..

[57] Gazzano O., Chambon M., Ferrec Y., Druart G. (2024). Long-Term Radiometric Stability of Uncooled and Shutterless Microbolometer-Based Infrared Cameras. Sensors.

[58] Brazane S., Riou O., Delaleux F., Ibos L., Durastanti J.F. (2025). Management of thermal drift of bolometric infrared cameras: Limits and recommendations. Quant. InfraRed Thermogr. J..

[59] Lin D., Cui X., Wang Y., Yang B., Tian P. (2022). Pixel-wise radiometric calibration approach for infrared focal plane arrays using multivariate polynomial correction. Infrared Phys. Technol..

[60] Tattersall G.J. (2016). Thermimage: Thermal Image Analysis.

[61] Harvey P. ExifTool by Phil Harvey: FLIR File Format Tags. https://exiftool.org.

[62] Pušnik I., Geršak G. (2021). Evaluation of the Size-of-Source Effect in Thermal Imaging Cameras. Sensors.

[63] Mendez-Bohorquez M.-D., Ebert J., Sommerlade L., Schmoll R., Kroll A. (2026). The size of source effect for middle- and long-wavelength infrared cameras: Assessment by direct measurements and a modulation transfer function approach. Quant. InfraRed Thermogr. J..

[64] Dziarski K., Hulewicz A., Dombek G. (2021). Lack of Thermogram Sharpness as Component of Thermographic Temperature Measurement Uncertainty Budget. Sensors.

[65] Hulewicz A., Dziarski K., Dombek G. (2021). The Solution for the Thermographic Measurement of the Temperature of a Small Object. Sensors.

[66] Lee H., Kang M.G. (2023). Infrared Image Deconvolution Considering Fixed Pattern Noise. Sensors.

[67] Ramesh J., Loney T., Du Plessis S., Rivas H., Sagahyroon A., Aloul F., Boillat T. (2026). Machine Learning Calibration of Smartphone-Based Infrared Thermal Cameras: Improved Bias and Persistent Random Error. Sensors.

[68] Ukiwe C.C., Adeshina S.A., Tsado J., Adetokun B.B. (2024). Infrared Thermal Image Dataset of High Voltage Electrical Power Equipment Under Different Operating Conditions.

[69] Ukiwe C.C., Adeshina S.A., Jacob T., Adetokun B.B. (2024). Deep learning model for detection of hotspots in high voltage electrical power equipment using infrared thermal images. J. Electr. Syst. Inf. Technol..

[70] Han S., Dong J., Huang Y., Zhang B. (2026). An Intelligent Multi-Emissivity Infrared Temperature Correction Method for Substation Equipment Based on Semantic Segmentation. Sensors.

[71] (2018). Standard Practice for Measuring and Compensating for Emissivity Using Infrared Imaging Radiometers.

[72] Sun D., You E., Zhang T., Xu J., Wang X., Ren X., Tao W. (2024). A review of thermal contact conductance research of conforming contact surfaces. Int. Commun. Heat Mass Transf..

[73] Krapez J.-C. Multiwavelength Thermometry without a priori Emissivity Information: From Promise to Disillusionment. Proceedings of the SPIE 13470, Thermosense: Thermal Infrared Applications XLVII.

[74] Koo T.K., Li M.Y. (2016). A Guideline of Selecting and Reporting Intraclass Correlation Coefficients for Reliability Research. J. Chiropr. Med..

[75] Schmid J., Bicat D., Elfner M., Bauer H.-J. (2021). Improved in-situ calibration applied to infrared thermography under high angles of view. Infrared Phys. Technol..

[76] Zhang P., Li Y., Li Y., Chen G., Hua W. (2022). A Proposition to Correct Infrared Emissivity Measurements for Curved Surface Targets Based on the Irradiation Reflection Method. Photonics.

[77] Wang Y., Sugawara M. (2026). Extension and Method-to-Method Agreement Assessment of a Visible-Image-Assisted Thermal Imaging Method for Directional Longwave Radiation Characterization of Building Heating Equipment. Buildings.

[78] Lavielle N., Othman A.M., Hervé A., Hamaoui G., Fei J., Tan J.Y., Marty F., Li H., Mongruel A., Beysens D. (2024). Infrared Spectral Emissivity Dynamics of Surfaces Under Water Condensation. Adv. Funct. Mater..

